# Surgical management and postoperative imaging assessment of hallux valgus

**DOI:** 10.1007/s00256-026-05327-8

**Published:** 2026-08-08

**Authors:** Hicham Bouredoucen

**Affiliations:** Division of Radiology, Department of Imaging and Medical Informatics, Geneva University Hospitals, University of Geneva, Rue Gabrielle-Perret-Gentil 4, 1211 Geneva 14 Geneva, Switzerland

**Keywords:** Hallux valgus, Weight-bearing computed tomography, Radiography, Osteotomy, Arthrodesis, Postoperative complications

## Abstract

Hallux valgus (HV) is a common and complex three-dimensional deformity of the first ray, resulting from multifactorial interactions involving anatomical, biomechanical, ligamentous, muscular, and extrinsic factors. The absence of a single etiological mechanism explains the wide variety of proposed surgical techniques and highlights the need for individualized management. The therapeutic strategy is based on a structured radioclinical weight-bearing assessment integrating angular parameters, first-ray stability, sesamoid alignment, and joint status. Depending on the severity of the deformity and the presence of associated instability, surgical options include distal, diaphyseal, or proximal osteotomies, Lapidus tarsometatarsal arthrodesis, and metatarsophalangeal arthrodesis. Certain complex situations, particularly those associated with metatarsus adductus or juvenile HV, require specific morphological analysis because of an increased risk of recurrence and particular biomechanical constraints. Postoperative imaging plays a central role in the assessment of surgical outcomes. It allows evaluation of deformity correction, osteotomy healing, fixation stability, sesamoid repositioning, and restoration of first-ray alignment. Weight-bearing radiographs remain the reference examination, whereas WBCT enables a more precise three-dimensional analysis of rotational correction, first metatarsal pronation, and the sesamoid complex. Imaging is also essential for the detection of postoperative complications, including recurrence, malunion, nonunion, avascular necrosis, transfer metatarsalgia, infection, osteoarthritis, and nerve injury. WBCT currently represents a major complementary tool for the analysis of multiplanar corrections and their biomechanical consequences. Thorough knowledge of normal and pathological postoperative findings is essential to optimize the management of HV.

## Introduction

Hallux valgus (HV) is one of the most common forefoot deformities, with an estimated prevalence ranging from 23 to 35.7% in the general population [1]. It corresponds to a complex three-dimensional deformity combining lateral deviation of the hallux, medial deviation and pronation of the first metatarsal, sesamoid malalignment, and progressive instability of the first ray [2]. Despite its high prevalence, no reference surgical technique has emerged, reflecting the multifactorial nature of this condition and the absence of a clearly identified single etiological mechanism [2].

The pathophysiology of HV results from complex interactions between extrinsic, anatomical, ligamentous, muscular, and biomechanical factors. Footwear, particularly high-heeled and narrow toe-box shoes, may contribute to progression of the deformity without constituting an isolated causal factor [2–4]. Several anatomical characteristics have also been implicated, including a relatively long first metatarsal, the morphology of the metatarsal head, and first-ray instability [5, 6]. Progressive imbalance of the periarticular soft tissues and tendons also contributes to maintenance and worsening of the deformity [7].

In this complex context, imaging plays a central role in both preoperative planning and postoperative assessment. Weight-bearing radiographs remain the standard examination for the analysis of angular parameters, sesamoid position, and first-ray alignment. Contemporary decision-making algorithms increasingly rely on radioclinical stratification integrating the hallux valgus angle (HVA), intermetatarsal angle (IMA), distal metatarsal articular angle (DMAA), as well as evaluation of the metatarsophalangeal (MTP) and tarsometatarsal (TMT) joints [8]. Depending on deformity severity and joint status, surgical options include distal, diaphyseal, or proximal osteotomies, Lapidus arthrodesis, and MTP arthrodesis [9–11].

Postoperative imaging is essential for evaluating the quality of correction, osteotomy healing, hardware stability, and biomechanical restoration of the first ray. In addition to conventional radiographs, weight-bearing computed tomography (WBCT) has emerged as a major tool enabling more precise three-dimensional analysis of rotational deformities and postoperative alignment, particularly regarding first metatarsal pronation and sesamoid realignment [12, 13]. Imaging also plays a fundamental role in the detection of postoperative complications such as recurrence, malunion, nonunion, avascular necrosis, infection, and transfer metatarsalgia [14–16].

The aim of this narrative review is to provide a synthesis of the surgical management strategies for HV and postoperative imaging assessment, with emphasis on the contributions of weight-bearing radiographs and WBCT in the analysis of surgical outcomes and postoperative complications.

## Multifactorial pathogenesis of hallux valgus and surgical implications

Understanding the multifactorial pathophysiology of HV is essential for interpreting its diverse anatomical presentations, understanding the rationale behind corrective procedures, and recognizing the expected postoperative imaging findings. HV arises from complex interactions among extrinsic, anatomical, and biomechanical factors, the relative contribution of which varies across patients [2].

Among the extrinsic factors, footwear has long been regarded as a major etiologic contributor. High-heeled shoes with a narrow toe box increase mechanical loading across the forefoot [4]. However, their incomplete penetrance and lack of association with juvenile HV suggest that they primarily promote progression of a pre-existing deformity rather than initiate it. Consequently, footwear is now considered a factor that modulates deformity progression rather than a primary cause [2, 17].

Anatomical characteristics of the first ray also contribute to the development of HV. A relatively long first metatarsal is more frequently observed in affected patients, whereas a rounder first metatarsal head may predispose to joint instability and increase the risk of recurrence following surgical correction, although morphological criteria remain insufficiently standardized [2, 18].

Progression of the deformity also results from progressive imbalance of the capsuloligamentous and musculotendinous structures. Lateral displacement of the flexor and extensor tendons, together with disruption of the balance between the abductor and adductor hallucis muscles, progressively perpetuates valgus deviation of the hallux [2, 19].

The role of biomechanical factors remains only partially established. Although sagittal hypermobility of the first ray remains controversial [6], recent evidence suggests that excessive coronal mobility and first-ray pronation play a more important role in deformity development [5]. Other abnormalities, including a tight Achilles tendon and pes planus, are frequently associated with HV but show no consistent relationship with deformity severity or the risk of postoperative recurrence [2, 6].

Regardless of its underlying cause, HV progresses through a relatively consistent sequence characterized by medial deviation of the first metatarsal, sesamoid displacement, valgus deviation of the proximal phalanx, lateral displacement of the flexor and extensor tendons, pronation of the first ray, and medial capsular insufficiency, ultimately resulting in a complex three-dimensional deformity [2]. Recognition of these components is fundamental to understanding the objectives of contemporary surgical techniques and interpreting the expected postoperative anatomical changes.

The heterogeneity of the underlying pathophysiological mechanisms explains the absence of a universally applicable surgical strategy and the coexistence of numerous corrective techniques targeting, according to the individual deformity, osseous abnormalities, soft-tissue imbalance, and biomechanical dysfunction [2]. For the musculoskeletal radiologist, understanding these mechanisms is essential for accurate postoperative assessment, evaluation of correction adequacy, and identification of complications or recurrent deformity.

## Imaging-guided surgical decision-making in hallux valgus

Weight-bearing imaging plays a central role in the surgical planning of HV, enabling accurate quantification of the deformity, identification of anatomical and articular abnormalities that guide surgical decision-making, and providing the reference standard for postoperative assessment. The hierarchical algorithm proposed by Stefan Wanivenhaus is based on a sequential evaluation that prioritizes the condition of the first MTP and first TMT joints before interpretation of angular measurements [8]. The principal imaging findings guiding selection of the surgical technique are summarized in Table 1. 

**Table 1:** Imaging-guided radioclinical algorithm for surgical decision-making in hallux valgus

Radioclinical assessment	Key imaging findings	Interpretation	Preferred surgical strategy
Metatarsophalangeal (MTP) joint status	Advanced MTP osteoarthritis (Coughlin–Shurnas stage 3–4 or Kellgren–Lawrence ≥ 3)	Non-reconstructible joint	First MTP arthrodesis, regardless of deformity severity
Tarsometatarsal (TMT) joint status	TMT instability and/or advanced osteoarthritis	Structural first-ray instability	Lapidus arthrodesis
Intermetatarsal angle (IMA)	≤ 15°	Mild first metatarsal deviation	Distal metatarsal osteotomy
	15–18/19°	Moderate deformity	Diaphyseal (e.g., Scarf) or proximal metatarsal osteotomy
	> 19°	Severe deformity; assess associated TMT instability	Proximal osteotomy or Lapidus arthrodesis, depending on TMT status
Hallux valgus angle (HVA)	< 15°, 15–40°, > 40°	Classifies overall deformity severity (mild, moderate, severe)	Complements surgical planning but should not be used as an isolated decision criterion
Functional assessment	MTP dorsiflexion ≥ 30°, functional range of motion > 50°, metatarsal index and load distribution	Evaluates forefoot function and predicts risk of transfer metatarsalgia	Guides adjunctive procedures and optimization of correction
Soft-tissue balance	Clinical and imaging evidence of periarticular soft-tissue imbalance	Persistent tendon and capsuloligamentous imbalance	Soft-tissue procedures performed in combination with osseous correction when indicated
Global radioclinical integration	Combined assessment of joint status, angular deformity, first-ray stability, and functional parameters	Defines the anatomical level of correction	Individualized surgical strategy

Surgical decision-making is based on a hierarchical radioclinical assessment prioritizing metatarsophalangeal and tarsometatarsal joint status before angular measurements. Imaging findings should always be integrated with clinical evaluation and first-ray function to guide individualized treatment

*HVA* hallux valgus angle, *IMA* intermetatarsal angle, *MTP* metatarsophalangeal, *TMT* tarsometatarsal

**Fig. 1:**
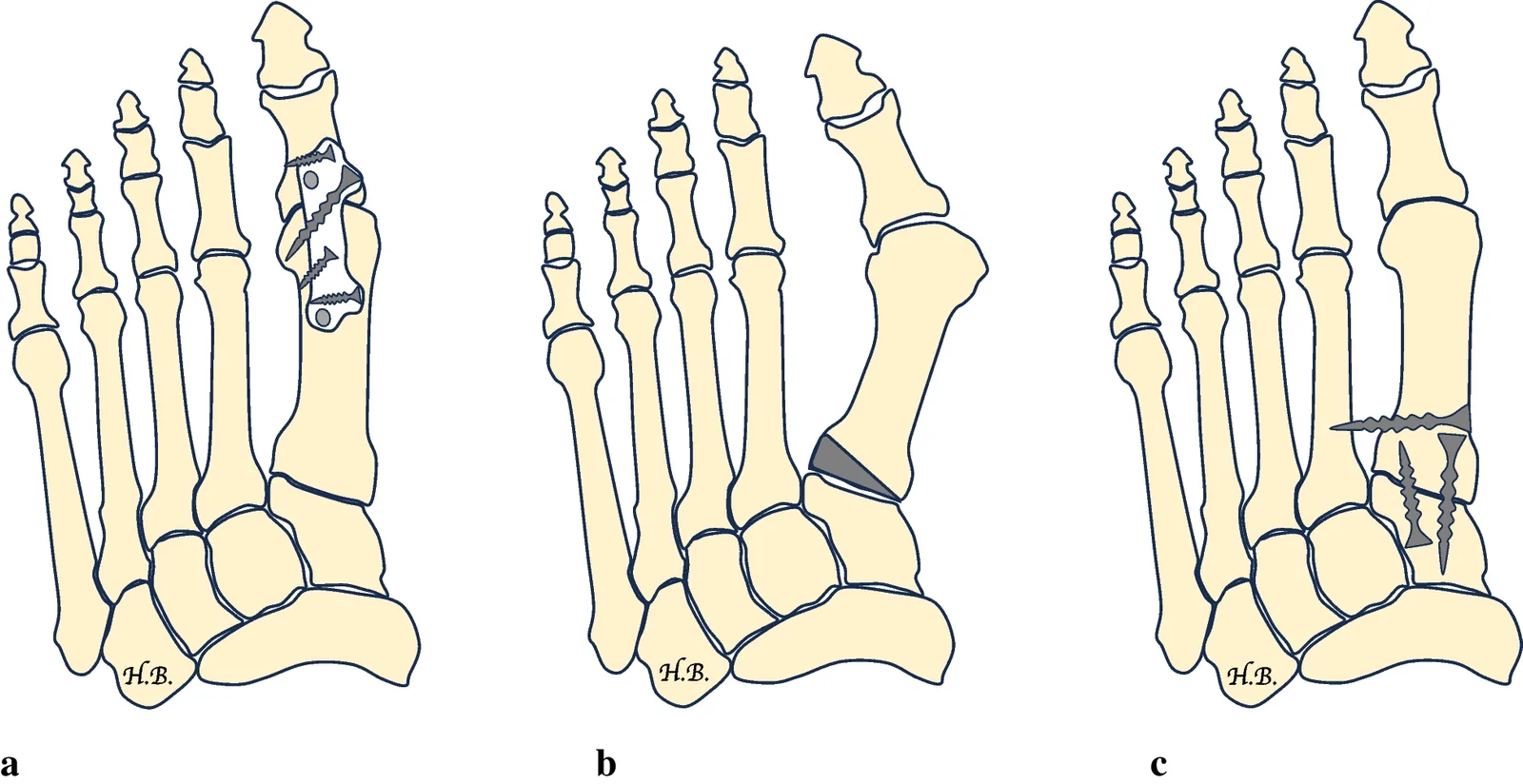
First-ray arthrodeses in the treatment of hallux valgus. **a** First metatarsophalangeal (MTP) arthrodesis indicated in advanced first MTP osteoarthritis (Coughlin and Shurnas stages 3–4 or Kellgren–Lawrence grades 3–4), regardless of the severity of angular deformity. The procedure aims to relieve pain and restore the weight-bearing function of the first ray. **b**,** c** Lapidus tarsometatarsal (TMT1) arthrodesis indicated in cases of instability or osteoarthritis of the tarsometatarsal complex. **b** It illustrates preparation of the joint surfaces with cartilage resection from the base of the first metatarsal and the medial cuneiform. **c** demonstrates TMT1 arthrodesis fixation using internal screw fixation following correction of first-ray alignment

The first step is to determine whether joint preservation is feasible. Advanced osteoarthritis of the first MTP joint (Coughlin and Shurnas grades 3–4 or Kellgren–Lawrence grade ≥ 3) is an indication for first MTP arthrodesis (Fig. 1), regardless of deformity severity [8]. Likewise, instability or advanced osteoarthritis of the first TMT joint favors a Lapidus arthrodesis (Fig. 1), which remains the reference procedure for first-ray instability [8]. This hierarchical approach underscores that joint pathology takes precedence over angular measurements in surgical decision-making.

**Fig. 2:**
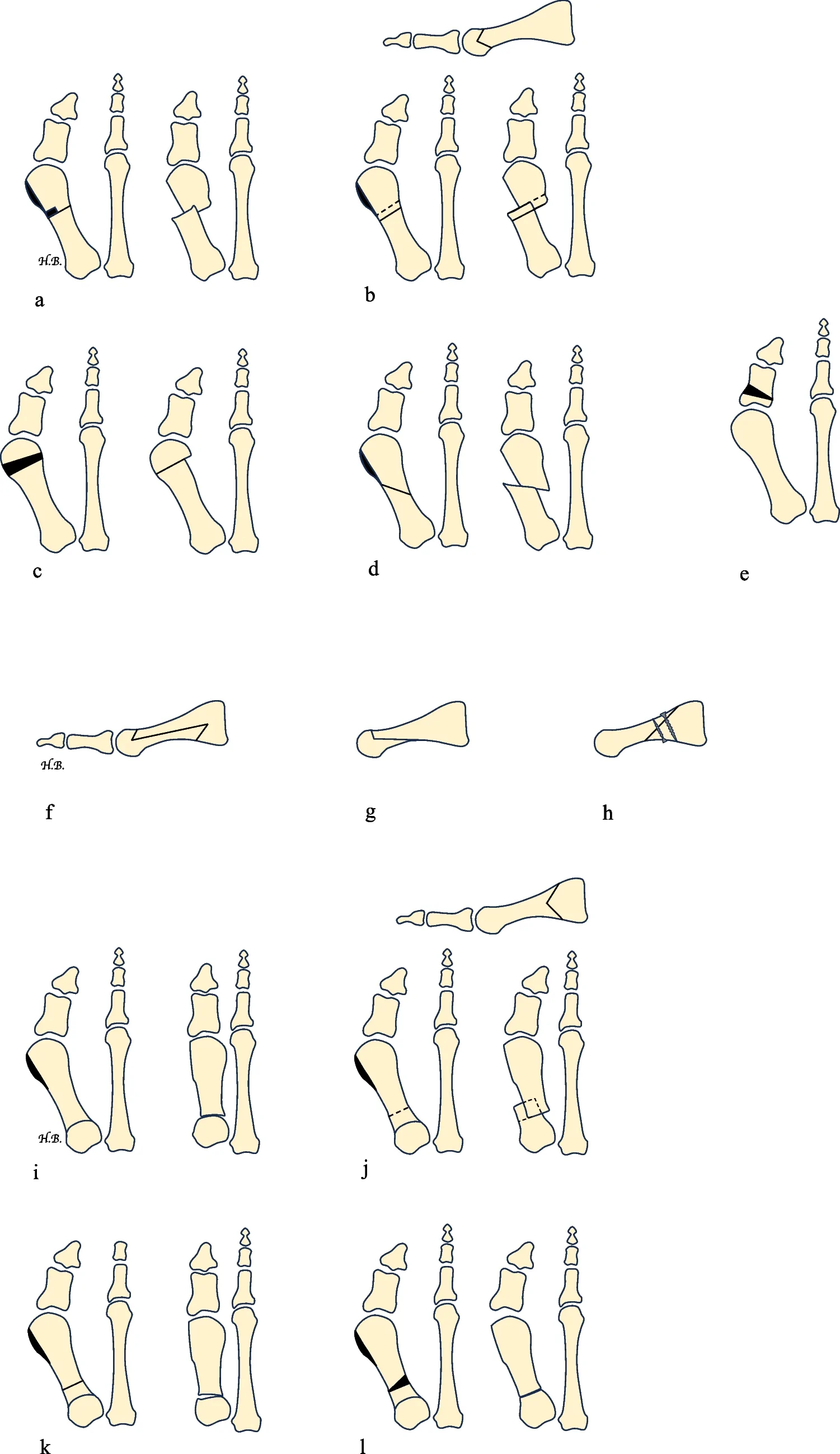
First metatarsal osteotomies in hallux valgus: classification according to anatomical level (distal, diaphyseal, and proximal). Distal first metatarsal osteotomies in the treatment of hallux valgus (**a**–**e**). Distal first metatarsal osteotomies are performed at the metatarsal neck and are primarily indicated for mild hallux valgus deformities in the presence of preservable metatarsophalangeal (MTP) and tarsometatarsal (TMT) joints. In imaging-based decision-making algorithms using weight-bearing assessment, they are generally reserved for deformities with an IMA ≤ 15°. They are frequently combined with lateral soft tissue release and/or an Akin osteotomy depending on the phalangeal component of the deformity. This figure illustrates the main distal techniques: **a** Mitchell osteotomy (step-cut osteotomy), **b** Chevron osteotomy (horizontal V-shaped osteotomy), **c** Hohmann osteotomy (closing wedge osteotomy, currently of mainly historical interest), **d** Wilson osteotomy (distal oblique osteotomy), and **e** Akin osteotomy—a proximal phalangeal osteotomy used as an adjunctive procedure rather than a metatarsal osteotomy, most commonly performed in combination with first metatarsal osteotomies. Diaphyseal first metatarsal osteotomies for hallux valgus correction (lateral view) (**f**–**h**). Diaphyseal osteotomies are indicated for moderate to severe deformities in the absence of non-preservable joint disease and without tarsometatarsal (TMT) instability. They allow multiplanar correction of the first metatarsal with precise control of alignment. From a radiologic perspective, they are generally proposed for intermediate IMA values, approximately between 15° and 19°. This figure illustrates the main diaphyseal techniques: **f** Scarf osteotomy, a Z-shaped osteotomy allowing controlled lateral translation of the distal fragment, frequently combined with an Akin osteotomy in more severe deformities; **g** extended Chevron osteotomy, an elongated V-shaped osteotomy extending into the diaphysis, allowing lateral translation of the metatarsal head with internal fixation; **h** Ludloff osteotomy, an oblique diaphyseal osteotomy allowing correction through rotation and translation around the fixation hardware. These techniques are often associated with medial exostectomy and lateral soft tissue release. Proximal first metatarsal osteotomies in the treatment of hallux valgus (**i**–**l**). Proximal first metatarsal osteotomies are performed at the metatarsal base, approximately 1–2 cm from the tarsometatarsal joint, and are primarily indicated for moderate to severe deformities when the joints are preservable and in the absence of TMT instability. They allow substantial correction of the intermetatarsal angle, particularly when markedly increased (approximately > 15–19°, depending on the case). This figure illustrates the main proximal techniques: **i** proximal crescentic osteotomy (convex crescent-shaped osteotomy), **j** proximal Chevron osteotomy, **k** proximal opening wedge osteotomy, and **l** proximal closing wedge osteotomy. These techniques are generally combined with medial exostectomy and lateral soft tissue release, with stabilization using internal fixation (screws, pins, or plates)

When both joints are preserved, radiographic parameters determine the appropriate level of correction. The IMA remains the primary determinant of osteotomy selection: ≤ 15° generally indicates a distal osteotomy; 15–18/19° favors a diaphyseal Scarf or proximal osteotomy; and > 19°, particularly when accompanied by suspected TMT instability, may warrant a Lapidus arthrodesis (Fig. 2) [8, 20, 21]. In contrast, the HVA is used primarily to stratify overall deformity severity (mild, < 15°; moderate, 15–40°; severe, > 40°) rather than as an isolated determinant of surgical strategy.

The assessment is further refined by functional evaluation of the first ray. First MTP dorsiflexion of at least 30° and a functional range of motion exceeding 50° are considered necessary for satisfactory forefoot function. In addition, the metatarsal index and plantar load distribution help predict the risk of transfer metatarsalgia and the potential need for adjunctive procedures.

**Fig. 3:**
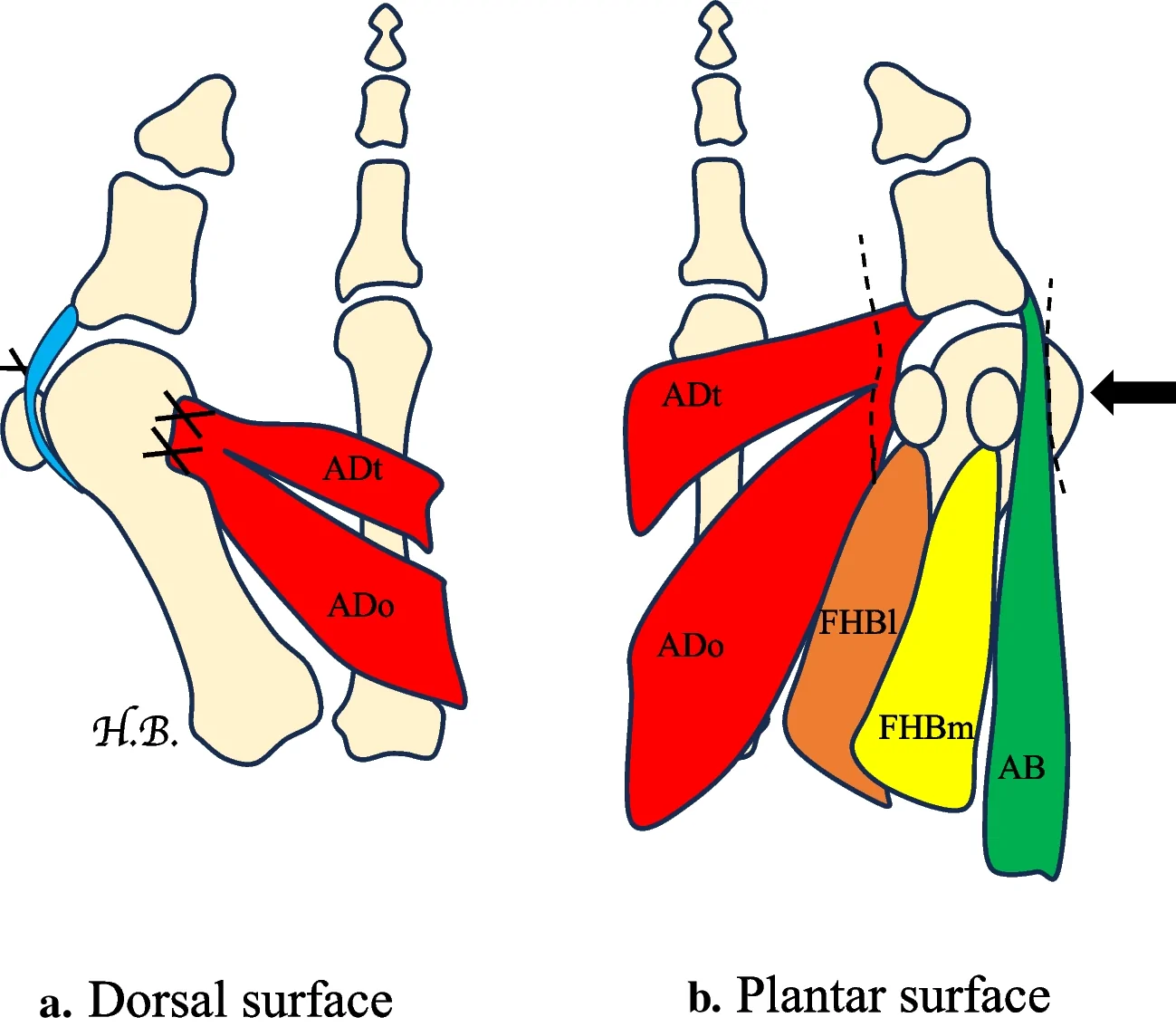
Distal soft tissue surgery. Medial capsule (blue). Medial osseous prominence (black arrow). On the medial side, the abductor hallucis tendon (green) inserts onto the medial sesamoid and the medial capsuloligamentous structures. On the lateral side, the oblique (Ado, red) and transverse (Adt, red) heads of the adductor hallucis insert onto the lateral sesamoid and the base of the proximal phalanx of the hallux. The lateral (FHBl, orange) and medial (FHBm, yellow) heads of the flexor hallucis brevis (FHB) insert onto the corresponding sesamoids. Distal soft tissue surgery, frequently combined with a metatarsal osteotomy or TMT arthrodesis, aims to correct hallux valgus by releasing contractures and retensioning the structures surrounding the MTP joint. Technically, the procedure includes the following: **a **a lateral step involving release of contracted structures (adductor tendon, deep transverse ligament, and capsule) and muscular rebalancing and **b **a medial step involving capsulotomy, resection of the medial osseous prominence, and capsular repair to realign the hallux. The objective is to restore joint balance while avoiding excessive dissection that could result in hallux varus

This integrated radioclinical assessment establishes a direct correspondence between imaging abnormalities and the anatomical level requiring correction. Soft-tissue procedures (Fig. 3) are now performed primarily as adjuncts to osseous correction in order to restore periarticular soft-tissue balance [11, 22–24]. For the musculoskeletal radiologist, understanding this decision-making process is essential for identifying the procedure performed, evaluating the adequacy of correction, and distinguishing a satisfactory postoperative result from recurrent deformity or complications.

Overall, surgical planning relies on a hierarchical integration of anatomical, articular, angular, and functional findings, enabling prediction of the operative technique and facilitating accurate interpretation of the expected postoperative imaging appearance.

## Hallux valgus in complex anatomical contexts: metatarsus adductus and juvenile hallux valgus

HV may occur in specific anatomical settings, most notably in association with metatarsus adductus or in skeletally immature patients. Recognition of these conditions is important because they influence radiographic assessment, surgical planning, and the risk of postoperative recurrence.

### Hallux valgus associated with metatarsus adductus

**Fig. 4:**
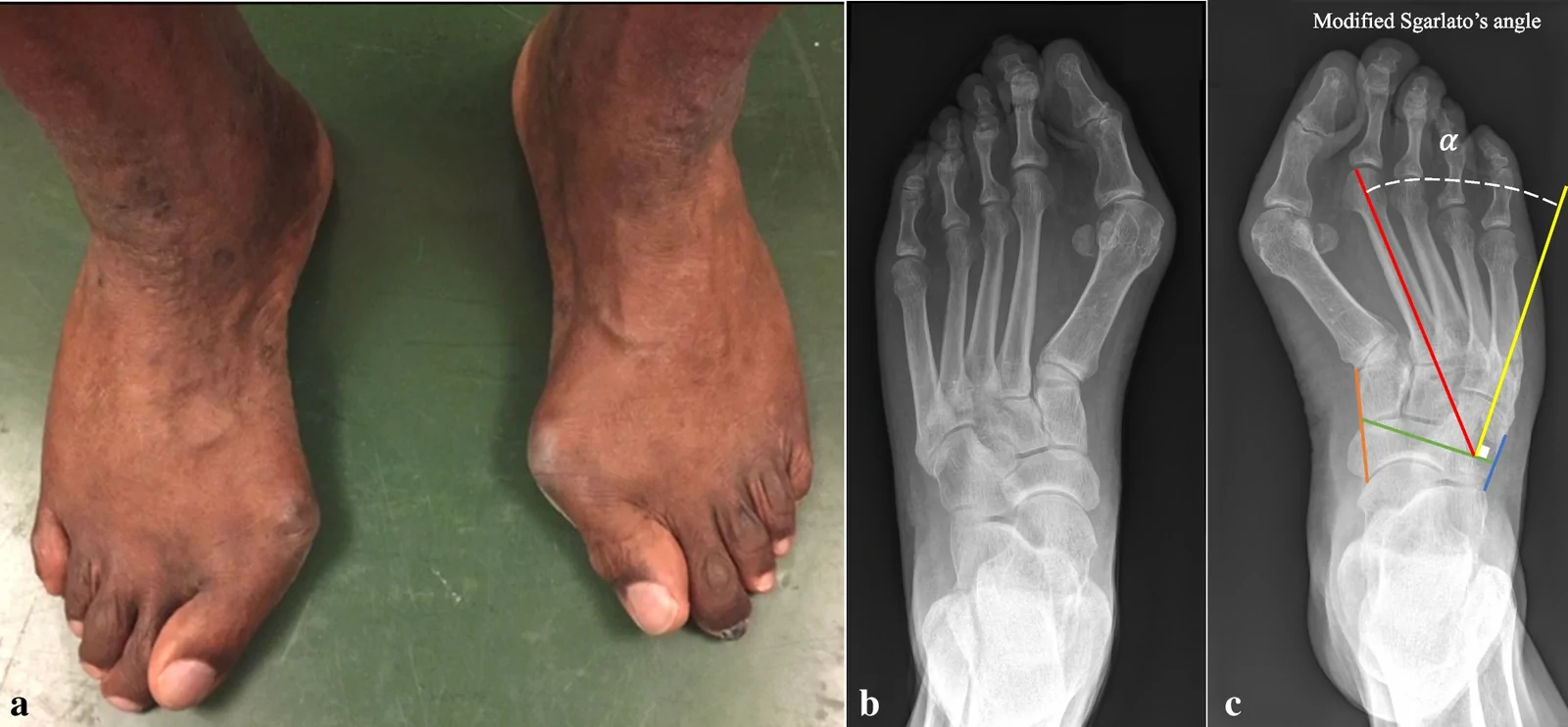
**a** Clinical evaluation of a patient with hallux valgus associated with metatarsus adductus. **b, c** Weight-bearing anteroposterior (AP) radiographs of the left foot and right foot. **c** Measurement of the modified Sgarlato angle. The lateral metatarsal axis is defined according to the following steps: (a) a first line (orange line) is drawn between the most medial point of the first-ray tarsometatarsal joint and the most medial point of the talonavicular joint; (b) a second line (blue line) connects the most lateral point of the fifth metatarsocuboid joint to the calcaneocuboid joint. A third line (green line) is then constructed by connecting the midpoints of lines A and B. From this line, a perpendicular line (yellow line) is drawn at 90° to the green line. The modified Sgarlato angle (*α*) corresponds to the angle formed between the yellow line and the longitudinal axis of the second metatarsal (red line). Recognition of metatarsus adductus is essential because it alters the interpretation of conventional hallux valgus measurements. Failure to identify this deformity may lead to underestimation of the global forefoot malalignment and a false impression of adequate correction based solely on the hallux valgus angle (HVA) and intermetatarsal angle (IMA), potentially contributing to postoperative recurrence. In complex or borderline cases, WBCT may provide complementary three-dimensional assessment of forefoot alignment and rotational deformities that cannot be reliably appreciated on conventional radiographs

Metatarsus adductus is characterized by medial deviation of the metatarsals in the transverse plane, whereas HV combines lateral deviation of the hallux with medial deviation of the first metatarsal (Fig. 4). Their coexistence is common, with metatarsus adductus increasing the risk of HV by approximately 3.5-fold and being present in nearly 30% of patients undergoing corrective surgery for HV [25, 26]. Metatarsus adductus is commonly classified according to the Bleck classification, which is based on forefoot flexibility and reducibility [27].

Radiographic evaluation relies on weight-bearing radiographs, including measurement of the HVA, IMA and metatarsus adductus angle. The modified Sgarlato angle, defined as the angle between the longitudinal axis of the second metatarsal and a perpendicular to the lateral tarsal border, is considered the reference standard because of its excellent reproducibility. A value greater than 20° is considered diagnostic of metatarsus adductus (Fig. 4) [25, 26].

Recognition of this association is essential because metatarsus adductus influences the interpretation of angular measurements, may lead to underestimation of deformity severity, and is associated with an increased risk of undercorrection and postoperative recurrence following HV surgery [26, 28].

### Juvenile hallux valgus

Juvenile HV is characterized by persistent open physes and residual skeletal growth potential, which contribute to a substantially increased risk of postoperative recurrence [29]. Its etiology is multifactorial and includes genetic predisposition, an increased DMAA, generalized ligamentous laxity, metatarsus adductus, and pes planovalgus [7, 16, 30, 31].

**Fig. 5:**
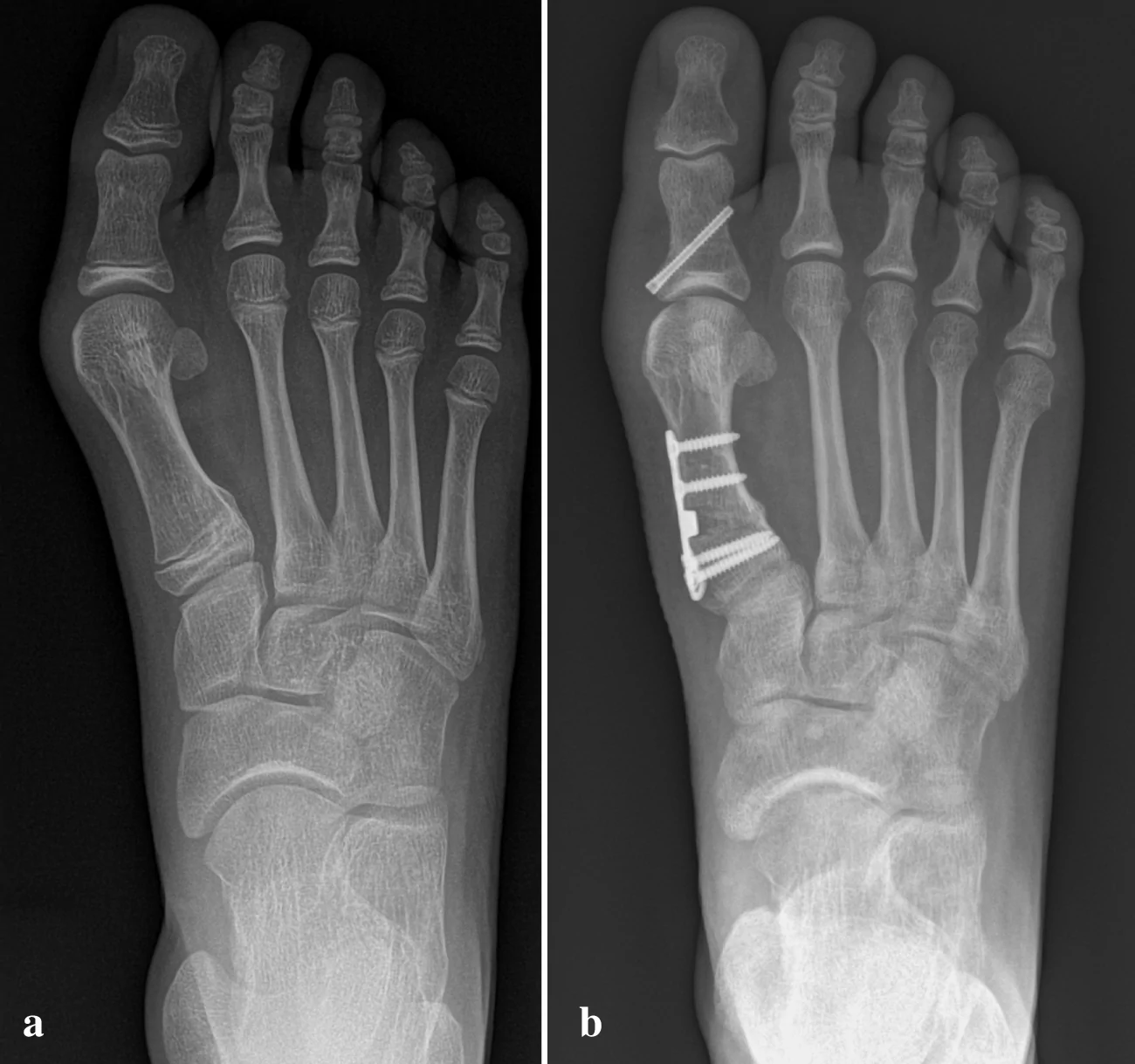
Juvenile hallux valgus of the right foot in a girl. Weight-bearing anteroposterior (AP) radiographs of the right foot obtained at 11 years of age (**a**). Corrective hallux valgus surgery was performed after skeletal maturity at 19 years of age (**b**). Persistent open physes should be systematically assessed, as failure to recognize skeletal immaturity may lead to underestimation of the risk of postoperative recurrence and premature surgical correction

Imaging assessment is based on weight-bearing anteroposterior and lateral radiographs (Fig. 5), allowing measurement of the HVA, IMA, DMAA, and interphalangeal angle, while also evaluating first-ray MTP joint congruency, sesamoid position, first-ray TMT joint alignment, and the status of the growth plates [17, 31]. Associated abnormalities, particularly metatarsus adductus, pes planovalgus, and first-ray TMT joint abnormalities, should be systematically assessed because of their influence on the risk of postoperative recurrence [29].

Surgical treatment is reserved for symptomatic patients and, whenever possible, should be postponed until skeletal maturity has been reached (Fig. 5) [31, 32]. The presence of open physes should be specifically reported on imaging, as it represents a well-established risk factor for postoperative recurrence [29].

## Normal postoperative imaging findings after hallux valgus surgery

### Standard postoperative radiographic findings

Postoperative assessment of HV primarily relies on weight-bearing radiographs, which remain the reference standard for evaluating the adequacy of surgical correction despite their inherent limitations in three-dimensional assessment. Interpretation begins with measurement of the principal angular parameters, including the HVA, IMA, and DMAA. A reduction in these measurements generally reflects satisfactory morphological correction, although their correlation with functional outcomes may be limited in complex cases [8]. The expected postoperative imaging findings according to the different surgical techniques are summarized in Table 2. 

**Table 2:** Expected postoperative imaging findings according to surgical technique

Surgical technique	Expected weight-bearing radiographic findings	WBCT added value	Normal postoperative evolution/key assessment points
Distal osteotomies (Chevron, minimally invasive techniques)	Decreased HVA and IMA, preservation of first metatarsal length, sesamoid recentering, congruent MTP joint, stable fixation	Assessment of residual first metatarsal pronation and sesamoid alignment	Progressive osteotomy healing, maintenance of correction, no secondary displacement or hardware failure
Diaphyseal osteotomies (Scarf)	Decreased HVA and IMA, restoration of first-ray alignment, improved MTP congruency	Three-dimensional evaluation of rotational correction and first-ray alignment	Progressive union, stable fixation, preservation of correction
Proximal osteotomies	Marked reduction of IMA with maintained first-ray alignment	Assessment of residual multiplanar deformity when radiographs are inconclusive	Progressive consolidation and maintenance of correction
Lapidus arthrodesis	Restoration of first-ray alignment, decreased HVA and IMA, TMT fusion, sesamoid recentering	Precise evaluation of TMT fusion, first metatarsal pronation correction, and medial column alignment	Progressive osseous fusion without loss of correction or hardware failure
First MTP arthrodesis	Solid osseous fusion, stable alignment, preserved hardware position	Evaluation of fusion and residual rotational alignment when required	Complete fusion with stable alignment and fixation

Weight-bearing radiographs remain the reference examination for routine postoperative follow-up. WBCT is complementary and is mainly indicated when conventional radiographs are inconclusive or when detailed three-dimensional assessment of rotational correction, sesamoid alignment, or osseous fusion is required

*DMAA* distal metatarsal articular angle, *HVA* hallux valgus angle, *IMA* intermetatarsal angle, *MTP* metatarsophalangeal, *TMT* tarsometatarsal, *WBCT* weight-bearing computed tomography

Assessment of the first ray is a key component of postoperative evaluation. The radiologist should assess its length, alignment, and position, while specifically looking for iatrogenic shortening or elevation that may alter forefoot load distribution [33, 34]. Recentering of the sesamoids beneath the first metatarsal head is an important indicator of adequate correction, as is restoration of first MTP joint congruency. Construct stability should also be evaluated by confirming the absence of secondary displacement, loss of correction, or hardware failure. 

**Fig. 6:**
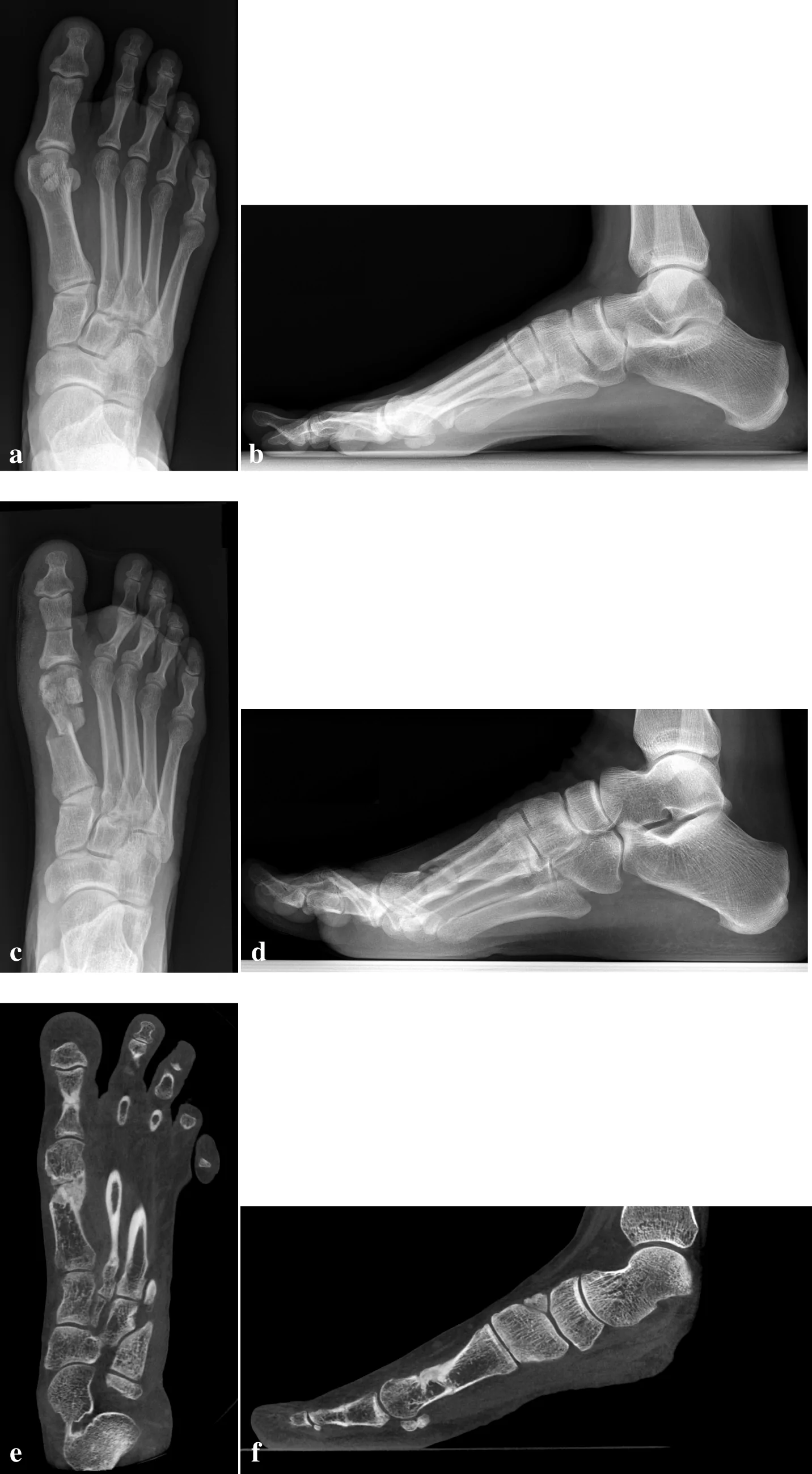
41-year-old woman with right hallux valgus. Preoperative weight-bearing anteroposterior (AP) (**a**) and lateral (**b**) radiographs. Follow-up weight-bearing AP (**c**) and lateral (**d**) radiographs after Scarf and Akin osteotomies demonstrate achieved union and maintenance of correction. Postoperative WBCT with axial (**e**) and sagittal (**f**) reconstructions confirms osseous union and provides a three-dimensional assessment of first-ray alignment under physiological loading conditions. Compared with conventional non-weight-bearing imaging, including radiographs and conventional CT, WBCT provides complementary information by enabling a more comprehensive assessment of osseous healing and first-ray alignment under mechanical loading conditions. Reliance solely on conventional angular measurements may underestimate residual alignment abnormalities despite apparently satisfactory correction

Expected postoperative findings vary according to the surgical technique. Distal and minimally invasive osteotomies typically result in decreased HVA and IMA while preserving first-ray length and restoring sesamoid alignment [35, 36]. Diaphyseal osteotomies provide three-dimensional correction and improve first MTP joint congruency (Fig. 6) [10, 37], whereas proximal osteotomies primarily address correction of the IMA. Following Lapidus arthrodesis, imaging should confirm restoration of first-ray alignment, successful first TMT joint fusion, and recentering of the sesamoids [38–41]. After the first MTP arthrodesis, complete osseous fusion and stable alignment are the expected imaging findings.

Comparison with the immediate postoperative radiographs is essential during follow-up to detect secondary loss of correction, hardware migration, or interval osseous displacement.

### Radiographic evolution of healing after hallux valgus osteotomies

Radiographic healing after HV surgery generally follows a reproducible pattern, although assessment criteria and follow-up protocols vary according to the surgical technique. Postoperative evaluation primarily relies on weight-bearing radiographs, which allow assessment of healing progression, osteotomy stability, and maintenance of the surgical correction.

Typical radiographic features of uncomplicated healing include progressive cortical bridging, gradual fading of the osteotomy line, absence of secondary displacement, and stable fixation hardware. Fourth-generation minimally invasive osteotomy techniques (META) demonstrate a well-described healing pattern, with approximately 17% radiographic union at 6 weeks, 70% at 3 months, and 90% at 6 months, corresponding to a mean time to union of approximately 13 weeks [42].

For distal osteotomies, including Chevron and Scarf procedures, radiographic follow-up is typically performed between 6 and 12 weeks, a critical interval for assessing bone union [43]. Proximal osteotomies generally demonstrate more rapid healing, whereas nonunion remains uncommon during this early postoperative period [44]. In contrast, arthrodesis procedures exhibit slower fusion kinetics than osteotomies and therefore require longer radiographic follow-up before definitive union can be confirmed [45].

In clinical practice, satisfactory radiographic union is generally expected between 6 and 12 weeks, with progressive confirmation by approximately 3 months postoperatively. Persistent visualization of the osteotomy line, absence of cortical bridging, or secondary loss of correction should raise suspicion for delayed union or nonunion. In these situations, WBCT may provide a more accurate assessment of osseous healing and assist in guiding subsequent management [42–45].

### Post-therapeutic imaging of hallux valgus associated with metatarsus adductus

Postoperative imaging assessment of HV associated with metatarsus adductus primarily relies on weight-bearing radiographs, which remain the reference standard for evaluating the adequacy of correction and its maintenance over time. Owing to the altered forefoot architecture in this anatomical setting, image interpretation should not be limited to conventional angular measurements but should also incorporate assessment of overall forefoot alignment [26].

Radiographic evaluation is based on several key morphological criteria [26]:

- Correction of the HVA and IMA.
- Realignment of the first ray toward a more physiological position. Although residual pronation or supination may be suspected on conventional radiographs, WBCT provides a more accurate assessment of first-ray rotational alignment.
- Widening of the first intermetatarsal space, reflecting satisfactory restoration of the relationship between the first and second metatarsals.
- Restoration of overall forefoot alignment, indicating harmonious correction without residual imbalance between the medial and lateral rays.

During postoperative follow-up, maintenance of correction is confirmed by the stability of these radiographic parameters. Conversely, worsening of angular measurements, deterioration of first-ray alignment, or persistence of metatarsus adductus should raise suspicion for secondary loss of correction or recurrent deformity, as this anatomical configuration is associated with an increased risk of long-term surgical failure [26, 28].

When radiographic assessment is inconclusive or the deformity is particularly complex, WBCT provides valuable complementary information by enabling three-dimensional evaluation of first-ray alignment, residual rotational deformity, and persistent forefoot malalignment.

### Normal postoperative imaging in juvenile hallux valgus

Weight-bearing radiographs remain the reference standard for the postoperative assessment of juvenile HV, allowing evaluation of both the adequacy of surgical correction and its maintenance during follow-up (Fig. 5) [29].

Radiographic evaluation is based on the assessment of the principal alignment parameters previously described, including the HVA, IMA, and DMAA, all of which should be measured on weight-bearing radiographs. The radiologist should also assess realignment of the first ray, recentering of the sesamoids beneath the first metatarsal head, and preservation of first metatarsal length to minimize alterations in forefoot load distribution. Although residual pronation or supination of the first ray may be suspected on conventional radiographs, WBCT provides a more accurate assessment of residual rotational alignment.

During postoperative follow-up, the radiologist should confirm maintenance of these radiographic parameters, with no secondary loss of correction, progressive increase in the HVA or IMA, or secondary displacement of the osseous fragments. Given the residual growth potential in skeletally immature patients, particular attention should be paid to the early radiographic detection of recurrent deformity during follow-up [29].

## Imaging of postoperative complications of hallux valgus

Postoperative complications following HV surgery remain relatively common despite the wide range of available surgical techniques. In a systematic review including more than 16,000 procedures, Barg et al. reported an overall patient dissatisfaction rate of approximately 10%, a recurrence rate of 5%, an incidence of transfer metatarsalgia of 6%, and nerve injury in 3% of cases, with complication rates varying according to the surgical technique employed [15].

Imaging plays a central role in the evaluation of failed correction, recurrent deformity, and osseous, mechanical, and soft-tissue complications. Weight-bearing radiographs remain the first-line imaging modality for assessing first-ray alignment. WBCT provides complementary three-dimensional assessment, particularly for the evaluation of rotational malalignment. Ultrasound is useful for assessing superficial soft-tissue and peripheral nerve complications and for guiding interventional procedures. Magnetic resonance imaging (MRI) is indicated when radiographic findings are inconclusive or when a more detailed evaluation of deep soft-tissue or osseous complications is required. The principal postoperative complications and their characteristic findings on radiography, WBCT, MRI, and ultrasound are summarized in Table 3.

**Table 3:** Postoperative complications after hallux valgus surgery: multimodality imaging findings

Complication	Radiographs (first-line)	WBCT	MRI	Ultrasound
Hallux valgus recurrence	Increased HVA and IMA, lateral sesamoid displacement, reopening of the first intermetatarsal space, loss of correction	Residual first metatarsal pronation, sesamoid malalignment, multiplanar deformity	Usually not indicated	—
Transfer metatarsalgia	Altered metatarsal parabola, first metatarsal shortening/elevation, loss of sesamoid coverage	Three-dimensional malalignment, malunion, rotational abnormalities	Bone marrow edema, MTP synovitis, stress fracture, plantar plate injury	—
Avascular necrosis	Progressive subchondral sclerosis, fragmentation, collapse, secondary osteoarthritis	Extent of collapse and residual deformity	Early marrow signal abnormalities, subchondral fracture, osteochondral collapse	—
Delayed union/Nonunion	Persistent osteotomy line, absent cortical bridging, sclerosis, secondary displacement	Cortical continuity, union status, hardware assessment	Rarely indicated	—
Malunion	Shortening, elevation, sagittal malalignment	Three-dimensional shortening, rotational deformity, sesamoid position, troughing	May demonstrate secondary overload	—
First MTP osteoarthritis	Joint-space narrowing, osteophytes, subchondral sclerosis, incongruity	Residual malalignment, overload, deformity	Cartilage loss, subchondral edema, synovitis	—
Infection	Late osteolysis, cortical erosion, hardware loosening	Cortical destruction, sequestrum, deep collections	Osteomyelitis, septic arthritis, soft-tissue abscess, sinus tract	Superficial collections, image-guided aspiration
Nerve injury/First web-space neuralgia	Normal	Normal	T2 hyperintense nerve, scar tissue	Nerve thickening, neuroma, fascicular disorganization
Hallux valgus with metatarsus adductus	Persistent forefoot malalignment, recurrent deformity	Persistent multiplanar deformity, rotational malalignment	Rarely indicated	—
Juvenile hallux valgus recurrence	Progressive increase in HVA/IMA, loss of correction during growth	Assessment of residual rotational deformity when needed	Rarely indicated	—

Radiographs are the first-line imaging modality for postoperative assessment. WBCT is mainly indicated for complex or recurrent deformities requiring three-dimensional evaluation. MRI is reserved for occult osseous or soft-tissue complications, whereas ultrasound is primarily useful for superficial infectious collections and peripheral nerve abnormalities

*HVA* hallux valgus angle, *IMA* intermetatarsal angle, *MTP* metatarsophalangeal, *MRI* magnetic resonance imaging, *WBCT* weight-bearing computed tomography

On MRI, distinguishing normal early postoperative changes from infection may be challenging. Isolated bone marrow edema is nonspecific and should not be interpreted in isolation. T1-weighted sequences are the most specific for detecting bone marrow involvement, whereas fluid-sensitive sequences, including STIR and fat-suppressed T2-weighted imaging, are the most sensitive for identifying marrow edema and inflammatory changes. In contrast, replacement of the normal fatty marrow signal on T1-weighted images associated with a fluid collection, cellulitis, a sinus tract, or adjacent soft-tissue involvement strongly increases the likelihood of osteomyelitis. MRI findings should always be interpreted in conjunction with the clinical presentation and laboratory results [46, 47].

Orthopedic hardware may produce susceptibility artifacts that limit evaluation of the operative site. Artifact reduction can be achieved using fast spin-echo sequences, fat suppression, increased receiver bandwidth, and dedicated metal artifact reduction techniques such as Slice Encoding for Metal Artifact Correction (SEMAC) and Multiacquisition Variable-Resonance Image Combination (MAVRIC), thereby improving diagnostic image quality [48, 49].

Intravenous gadolinium administration is not routinely required but improves the detection and characterization of deep infectious complications, particularly abscesses, sinus tracts, and septic arthritis, especially when surgical management is being considered.

### Recurrent hallux valgus

Recurrent HV is generally defined by a HVA > 20° associated with an IMA > 10°, although more stringent radiographic thresholds have also been proposed [14, 18, 50, 51]. Its reported incidence varies considerably depending on the definition used, the surgical technique, and the duration of follow-up. Recurrence rates are generally lower after distal Chevron osteotomy, higher after proximal osteotomies or resection procedures, and lowest following first-ray arthrodesis [14]. Reported recurrence rates range from approximately 10% to more than 60% across published series [43, 52].

Recurrence is multifactorial, resulting from a combination of morphological and biomechanical factors—including first-ray hypermobility, pes planovalgus, metatarsus adductus, and initial deformity severity—as well as factors related to the adequacy of surgical correction. Residual pronation of the first metatarsal, frequently associated with a persistent “round sign” and incomplete sesamoid reduction, has emerged as a major predictor of recurrent deformity [18, 50, 52–54].

**Fig. 7:**
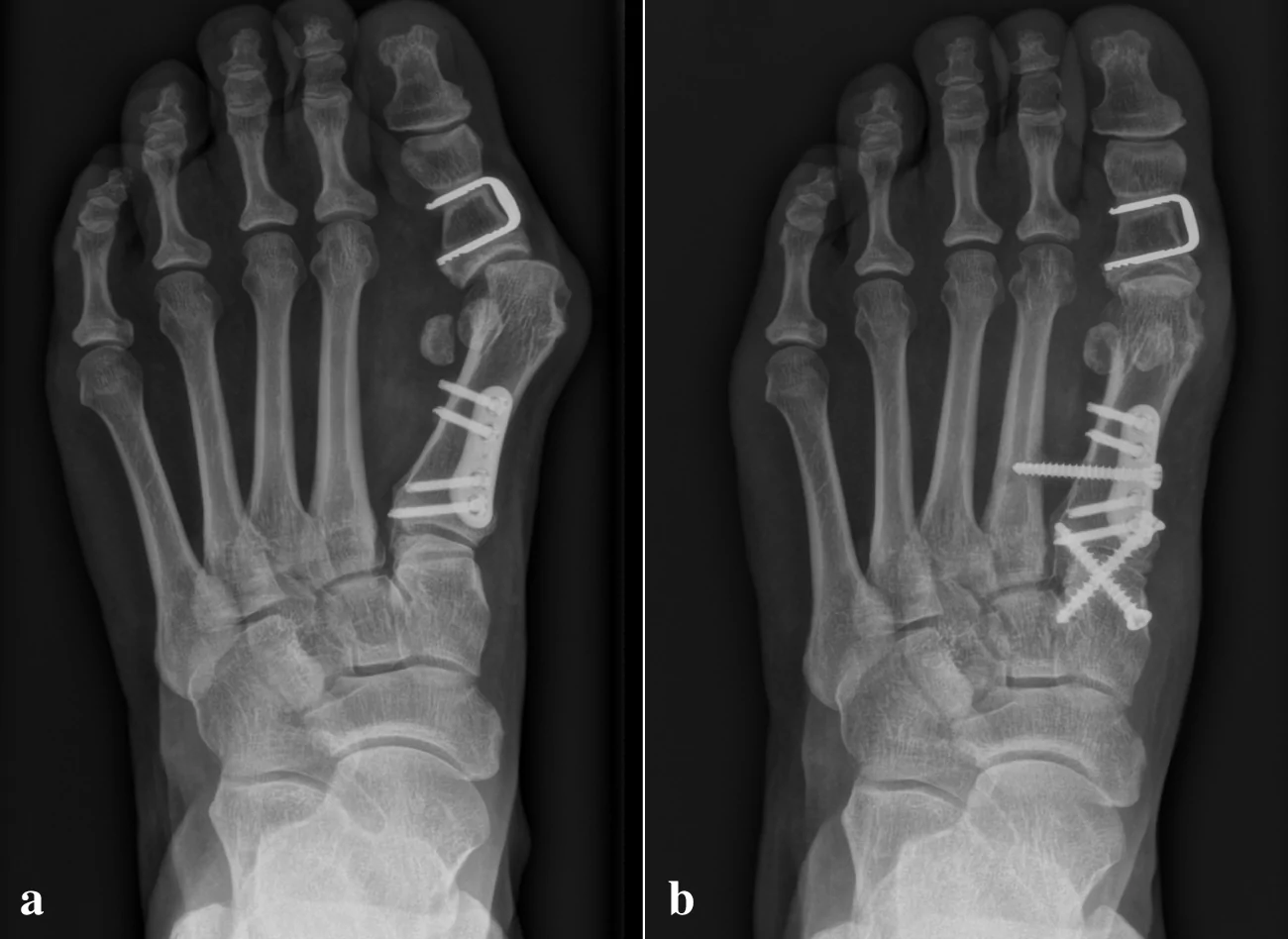
A 46-year-old woman, left foot. Recurrence of hallux valgus following proximal first metatarsal base osteotomy and proximal phalanx osteotomy of the hallux. Weight-bearing anteroposterior (AP) radiographs (**a**,** b**). Radiograph (**a**) demonstrates recurrence of hallux valgus, mainly related to first-ray hypermobility, associated with pain and transfer metatarsalgia, subsequently treated with Lapidus TMT1 arthrodesis (**b**). Comparison with the immediate postoperative radiographs is essential to distinguish true recurrence from insufficient initial correction, as failure to do so may lead to underestimation of secondary loss of correction

Evaluation primarily relies on weight-bearing radiographs, which allow comparison between immediate postoperative and follow-up examinations [14, 55]. Recurrence is characterized by a progressive increase in the HVA and IMA, lateral migration of the sesamoids, widening of the first-ray intermetatarsal space, and secondary loss of correction (Fig. 7). Comparison with the immediate postoperative radiographs is essential to distinguish inadequate initial correction from true recurrent deformity.

WBCT is particularly valuable when recurrence is suspected or before revision surgery. It enables three-dimensional assessment of first-ray metatarsal pronation, sesamoid alignment, and multiplanar malalignment, providing information that cannot be obtained from conventional radiographs [14, 18].

Accordingly, imaging plays a pivotal role in identifying the underlying mechanism of recurrence and in guiding preoperative planning for complex revision procedures [14, 56–58].

### Transfer metatarsalgia

Transfer metatarsalgia is characterized by pain beneath the lesser metatarsal heads resulting from mechanical insufficiency of the first ray following HV surgery. Its reported incidence ranges from 11 to 20% after certain osteotomies, particularly the Mitchell procedure [59, 60]. It most commonly results from biomechanical imbalance leading to overload of the lateral metatarsals, typically secondary to first metatarsal shortening, recurrent deformity, or malunion with elevation or dorsiflexion of the first metatarsal [61–63]. Clinically, patients present with pain beneath the lesser metatarsal heads, frequently associated with plantar callosities [61].

**Fig. 8:**
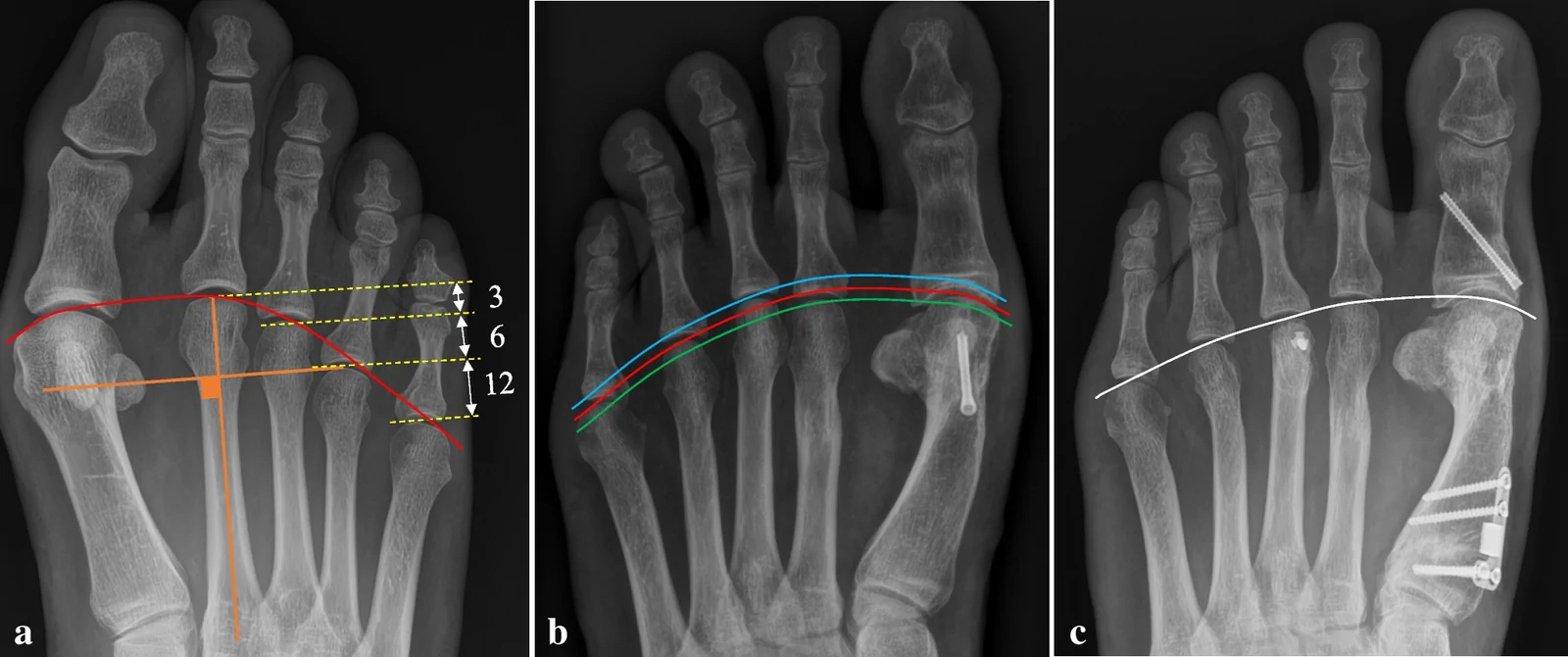
Assessment of the distal metatarsal parabola (Lelièvre parabola) on weight-bearing dorsoplantar radiographs. The distal metatarsal parabola is evaluated by analyzing the curve connecting the distal articular surfaces of the five metatarsal heads, which should form a single, smooth, continuous arc. According to Maestro et al. [64], assessment is based on two criteria: alignment of the center of the lateral sesamoid with the center of the fourth metatarsal head (SM4 line) and harmonious progression of the offsets between the lateral metatarsal heads, classically measuring approximately 3 mm between the second and third metatarsal heads, 6 mm between the third and fourth metatarsal heads, and 12 mm between the fourth and fifth metatarsal heads. The curves superimposed on the radiographs illustrate the metatarsal parabola: a single continuous arc indicates a harmonious parabola, whereas multiple arcs indicate disruption of the metatarsal parabola. **a** Weight-bearing dorsoplantar radiograph of the right foot in a 32-year-old woman demonstrating a harmonious distal metatarsal parabola with a single continuous arc, appropriate SM4 alignment, and the physiological 3-, 6-, and 12-mm offsets between the lateral metatarsal heads. **b** Weight-bearing dorsoplantar radiograph of the left foot in a 44-year-old man with transfer metatarsalgia following hallux valgus surgery. Excessive shortening of the second metatarsal disrupts the metatarsal parabola, resulting in multiple metatarsal arcs. **c** Postoperative weight-bearing dorsoplantar radiograph of the same patient following a third metatarsal Weil osteotomy, demonstrating restoration of a harmonious distal metatarsal parabola with re-establishment of a single continuous metatarsal arc

Weight-bearing radiographs are the first-line imaging modality. They are used to assess the metatarsal parabola, the relative lengths of the metatarsals, and first-ray alignment [64, 65]. The distal metatarsal parabola (Lelièvre parabola) is evaluated on weight-bearing dorsoplantar radiographs and should form a single, smooth, progressively curved arc (Fig. 8a). According to Maestro et al., its assessment is based on two criteria: alignment of the center of the lateral sesamoid with the center of the fourth metatarsal head (SM4 line) and harmonious progression of the offsets between the lateral metatarsal heads (M2–M3, M3–M4, and M4–M5), classically measuring approximately 3, 6, and 12 mm, respectively [64]. Disruption of this harmonious configuration, resulting in the appearance of multiple metatarsal arcs, is suggestive of transfer metatarsalgia (Fig. 8b), whereas the objective of surgery is to restore a single, continuous metatarsal arc (Fig. 8c). The radiologist should also evaluate for iatrogenic shortening of the first metatarsal, first-ray elevation, incomplete sesamoid coverage, and systematically compare follow-up studies with the immediate postoperative radiographs to distinguish inadequate initial correction from secondary deformity, a distinction that is essential for planning revision surgery.

**Fig. 9:**
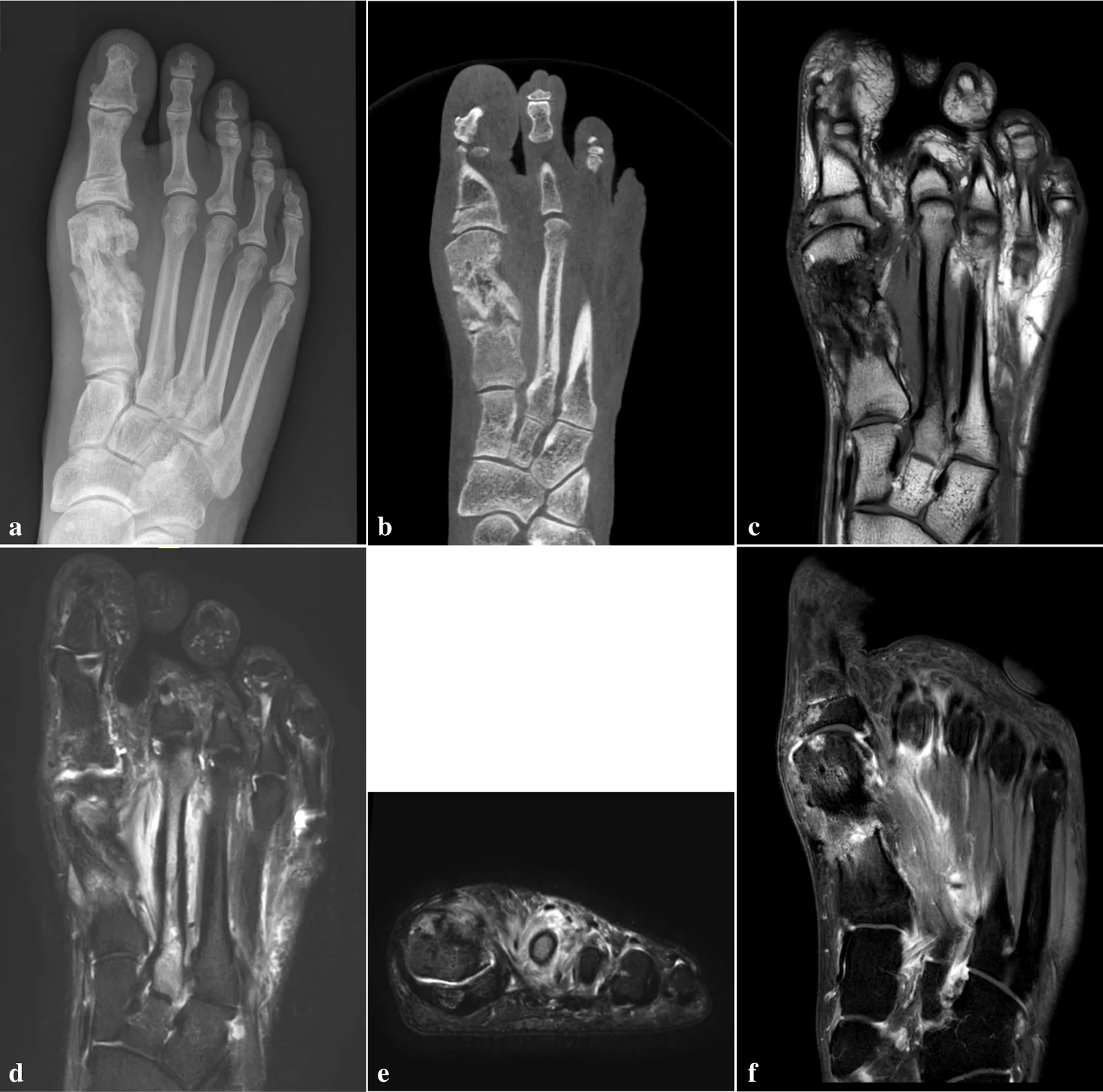
A 53-year-old man, right foot. Postoperative transfer overload following Scarf osteotomy for hallux valgus correction. Weight-bearing anteroposterior (AP) radiograph (**a**) and WBCT axial reconstruction (**b**) show no structural abnormality. Axial T1-weighted TSE imaging (**c**) does not demonstrate a clearly identifiable fracture line. Axial STIR (**d**) and coronal STIR (**e**) MR images demonstrate first metatarsophalangeal joint synovitis associated with bone marrow edema of the second metatarsal and adjacent soft tissues, consistent with transfer metatarsalgia complicated by a stress fracture secondary to postoperative biomechanical overload. Axial fat-suppressed post-gadolinium T1-weighted imaging (**f**) shows neither significant osseous enhancement nor soft-tissue collection suggestive of infection. This case illustrates that normal radiographs and WBCT do not exclude symptomatic postoperative complications. In the early postoperative setting, isolated bone marrow edema should not be interpreted as infection in the absence of T1 marrow replacement, abscess formation, or extensive soft-tissue involvement, thereby reducing the risk of false-positive diagnosis of osteomyelitis

Computed tomography, particularly WBCT, provides complementary information by characterizing three-dimensional alignment abnormalities, malunion, and rotational deformities responsible for the underlying biomechanical imbalance [61]. When radiographs and WBCT are unremarkable despite persistent symptoms, MRI is useful for demonstrating the consequences of chronic mechanical overload, including MTP synovitis, bone marrow edema, stress fractures of the lesser metatarsals—particularly the second metatarsal—and plantar plate injuries (Fig. 9).

### Avascular necrosis of the first metatarsal head

Avascular necrosis of the first metatarsal head is a rare complication, reported predominantly after distal osteotomies and less frequently following diaphyseal osteotomies [66–69]. It results from disruption of the vascular supply to the first metatarsal head secondary to the osteotomy, with the risk being substantially reduced when the plantar capsuloperiosteal blood supply is preserved [70–72].

Weight-bearing radiographs are the first-line imaging modality. Early radiographic findings include progressive subchondral sclerosis, which may subsequently progress to fragmentation and collapse of the first metatarsal head. Advanced stages are characterized by articular deformity with narrowing of the first MTP joint space and secondary osteoarthritis. Comparison with previous postoperative radiographs is essential to distinguish avascular necrosis from expected postoperative osseous remodeling.

MRI is the most sensitive modality for early diagnosis, detecting bone marrow abnormalities before morphologic changes become apparent on radiographs. In advanced stages, MRI demonstrates subchondral fractures and osteochondral collapse [16]. WBCT may provide complementary information by more accurately defining the extent of subchondral collapse and the residual osseous deformity.

The radiologist should recognize these imaging findings at an early stage, accurately determine the stage of disease progression, and assess the extent of articular involvement to facilitate appropriate clinical management.

### Nonunion and delayed union

Nonunion following metatarsal osteotomy is a rare complication, with a reported incidence of approximately 0.04%, which is even lower when TMT arthrodeses are excluded from analysis [15]. It occurs predominantly after proximal osteotomies because of greater mechanical stresses at the osteotomy site. The principal risk factors include inadequate fixation stability, smoking, malnutrition, and vitamin D deficiency [73–75].

**Fig. 10:**
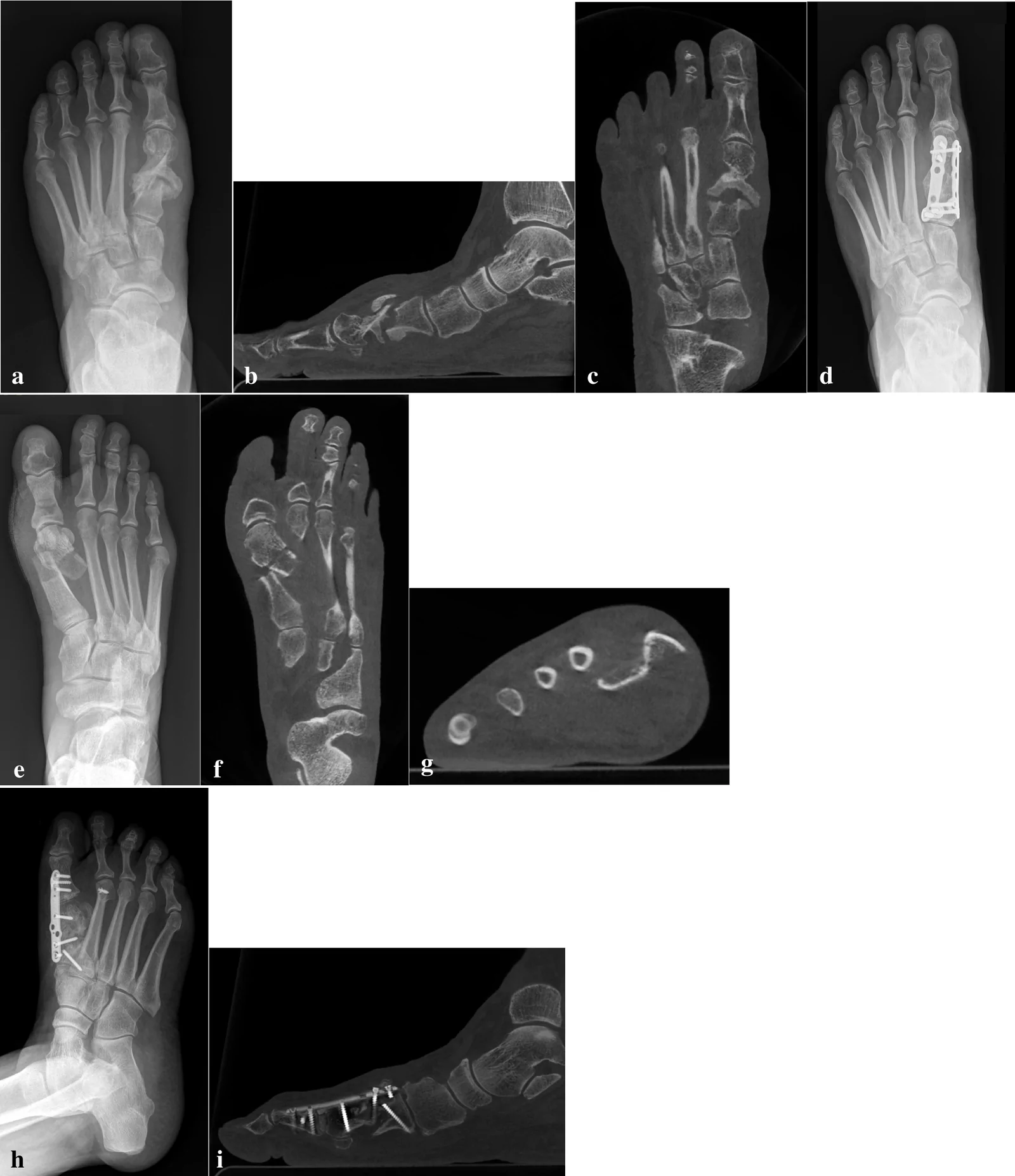
**a–d** A 54-year-old woman. Hallux valgus correction by Scarf osteotomy of the left foot. First metatarsal fracture and nonunion: **a **weight-bearing anteroposterior (AP) radiograph, **b **sagittal, and **c **axial WBCT reconstructions. Revision treatment with osteosynthesis: **d **follow-up weight-bearing AP radiograph. **e–g** Another patient, a 51-year-old woman. Status post hallux valgus correction of the right foot with Scarf and Akin osteotomies. Secondary fracture with displacement: **e** weight-bearing AP radiograph, **f** axial, and **g** coronal WBCT reconstructions. **h, i** A 62-year-old woman, right foot. **h** Non-weight-bearing oblique radiograph and **i** sagittal WBCT reconstruction. Status post Scarf osteotomy complicated by nonunion, proximal screw fracture, plate displacement, and shortening of the first metatarsal. No osseointegration of the bone graft is observed. These cases illustrate the complementary role of WBCT in the assessment of delayed union and nonunion after hallux valgus surgery. Compared with conventional non-weight-bearing CT, WBCT provides additional information by assessing bone healing and first-ray mechanical stability under physiological loading conditions. It may reveal subtle mechanical instability, secondary displacement, or loss of correction that can be underestimated on unloaded examinations, thereby reducing the risk of false-negative assessment before revision surgery. Conversely, the persistence of an osteotomy line or incomplete osseous bridging should not be considered diagnostic of nonunion in isolation, as this may lead to false-positive interpretations in cases of progressive healing

Weight-bearing radiographs are the first-line imaging modality. Findings suggestive of delayed union or nonunion include persistence of the osteotomy line, absence of cortical bridging, sclerosis of the osteotomy margins, secondary displacement, and lack of progressive osseous healing over time. Comparison with previous postoperative radiographs is essential to distinguish delayed union from established nonunion and to identify secondary loss of correction (Fig. 10).

WBCT is indicated when radiographic findings are equivocal or before revision surgery. It provides three-dimensional assessment of cortical continuity, osseous union, fixation stability, and any secondary displacement, thereby facilitating accurate diagnosis and preoperative planning.

### Malunion of metatarsal osteotomies

**Fig. 11:**
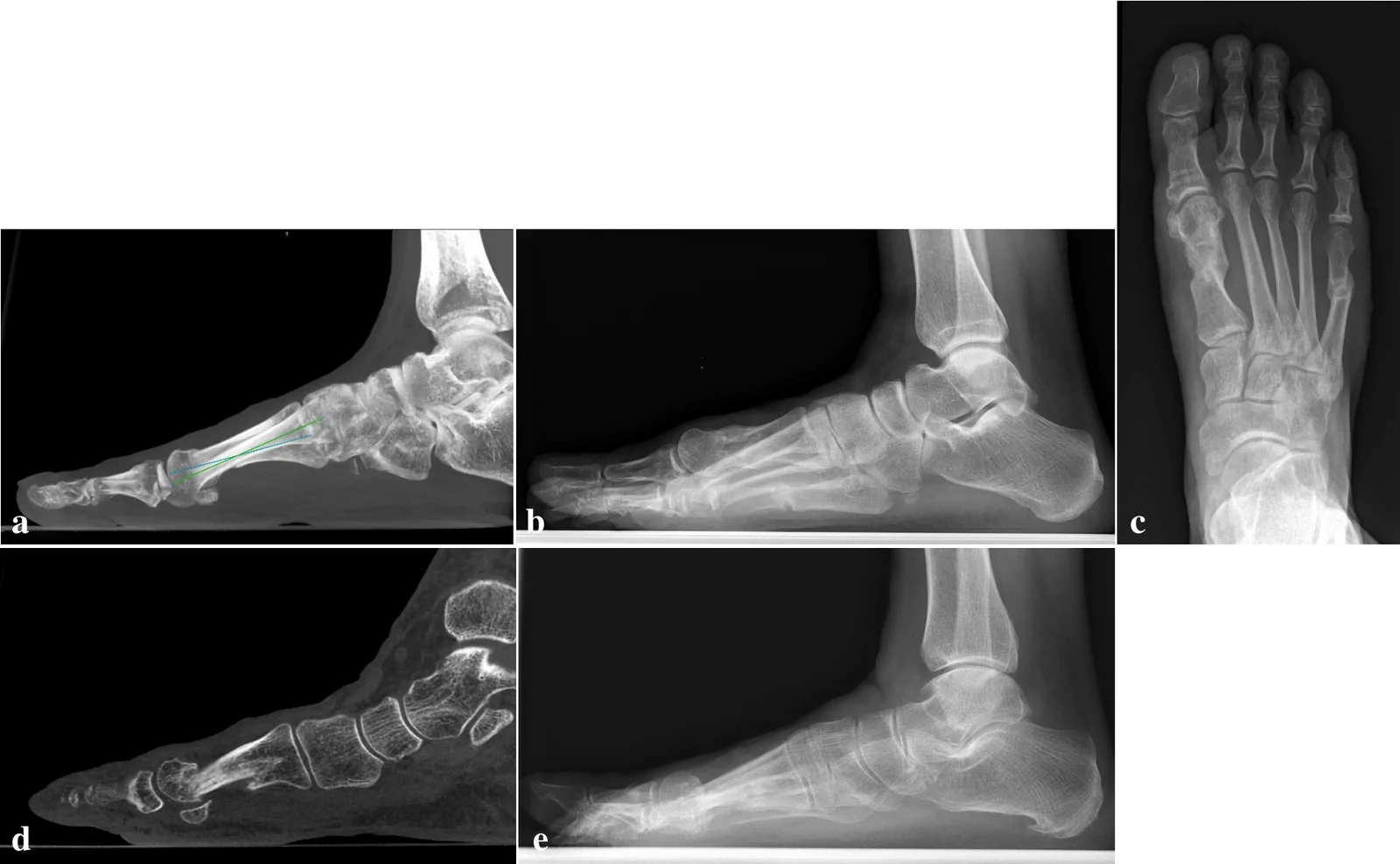
**a–c** Dorsiflexion malunion. **a** Three-dimensional volume-rendered reconstruction from WBCT of the right foot in a 47-year-old woman. The longitudinal axes of the first and second metatarsals are represented by a blue line (MT1) and a green line (MT2), respectively, drawn according to the medullary diaphyseal axis. The sagittal angle between these two axes demonstrates an 8° elevation of the first metatarsal, consistent with dorsiflexion malunion. This deformity is associated with first-ray shortening secondary to Scarf osteotomy and is responsible for severe transfer metatarsalgia. **b** Weight-bearing lateral and **c** anteroposterior (AP) radiographs. **d, e** Delayed union with dorsiflexion malposition. **d **WBCT sagittal reconstruction of the right foot in a 72-year-old woman. Status post Akin and Scarf osteotomies. **e** Weight-bearing lateral radiograph. These cases illustrate the complementary role of WBCT in the assessment of sagittal malalignment after hallux valgus surgery. By providing three-dimensional evaluation of first-ray alignment under physiological loading conditions, WBCT allows a more comprehensive assessment of deformities such as first metatarsal elevation and shortening, which may be underestimated on conventional imaging. Reliance solely on radiographic angular measurements may lead to false-negative interpretation of biomechanical abnormalities despite apparently satisfactory correction. Conversely, apparent sagittal malposition on radiographs alone should be interpreted cautiously because projectional factors may result in false-positive diagnosis of malunion

Malunion refers to osseous healing in a non-anatomical position following metatarsal osteotomy. It may involve abnormalities in the length, sagittal position, and rotational alignment of the first ray. First metatarsal shortening is a common consequence of osteotomy, with a reported incidence that varies according to the surgical technique employed [35, 76]. When excessive, shortening results in the first-ray insufficiency, leading to overload of the lesser metatarsals and subsequent plantar plate injury [61, 77]. Sagittal malalignment, including elevation or, less commonly, plantarflexion of the first metatarsal, alters forefoot load distribution and predisposes to MTP pain and secondary osteoarthritis (Fig. 11).

**Fig. 12:**
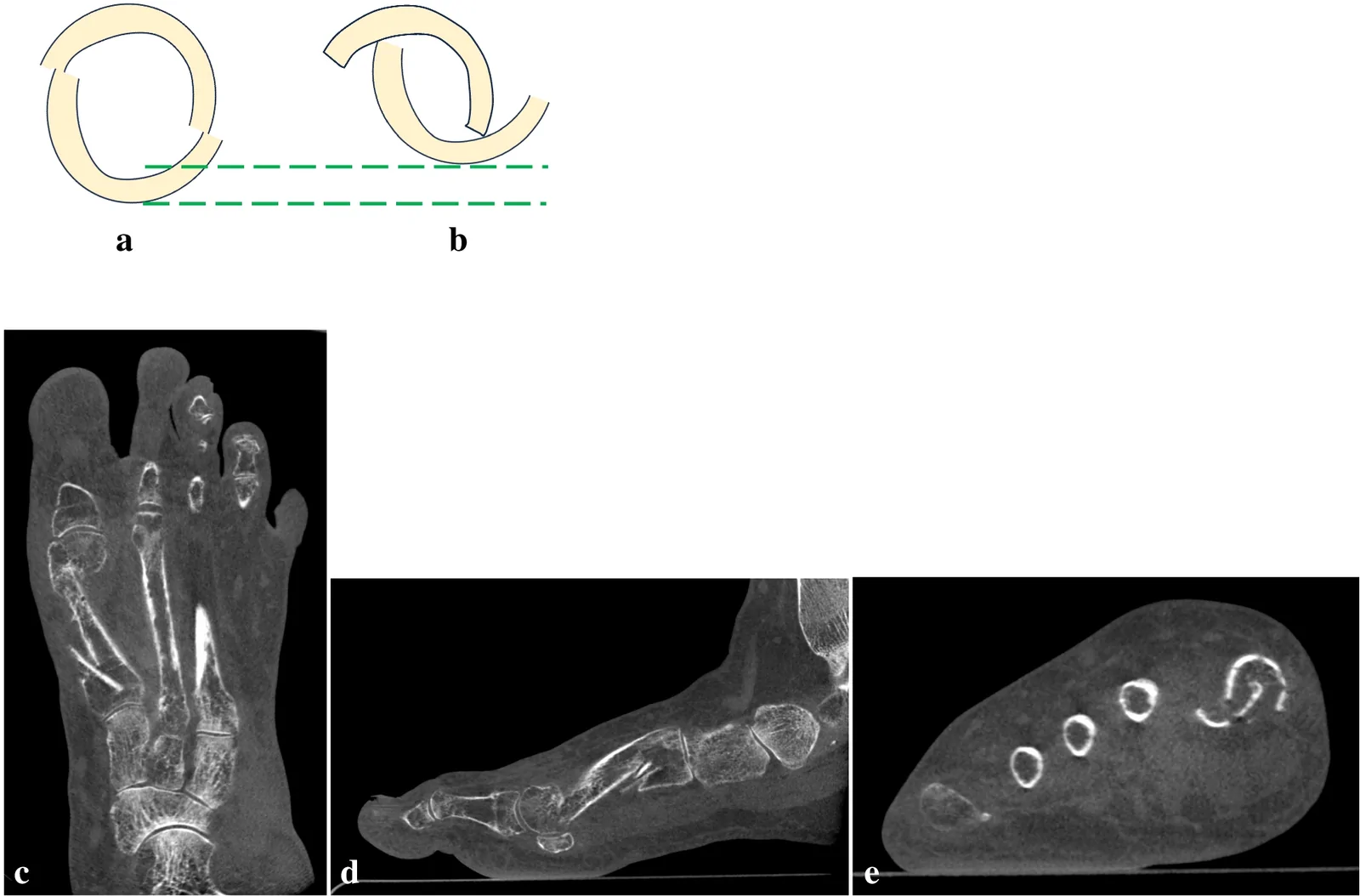
Scarf osteotomy and the “troughing” phenomenon. **a, b **Schematic drawings. The orientation of the osteotomy in the coronal plane determines the degree of plantar translation of the metatarsal head. **a** Example of a correctly performed Scarf osteotomy with preservation of cortical continuity and maintenance of metatarsal height. **b** Appearance of malunion with “troughing,” corresponding to cortical impaction into the medullary canal of the proximal metatarsal diaphysis, resulting in loss of height and relative elevation of the distal segment (dashed lines). Right foot, 63-year-old woman. **c–e **Scarf and Akin osteotomies complicated by troughing (cortical collapse). **c **WBCT axial, **d **sagittal, and **e** coronal reconstructions demonstrate cortical impaction, loss of first metatarsal height, and sagittal malalignment of the distal fragment. This case illustrates the complementary role of WBCT in the assessment of Scarf osteotomy-related malunion. Three-dimensional evaluation under weight-bearing conditions allows more accurate characterization of cortical impaction, loss of height, and first-ray alignment, which may be underestimated on conventional radiographs. Apparent loss of metatarsal height on isolated radiographic projections should be interpreted cautiously to avoid false-positive diagnosis of troughing, whereas subtle three-dimensional collapse may be missed on conventional imaging, leading to false-negative assessment

**Fig. 13:**
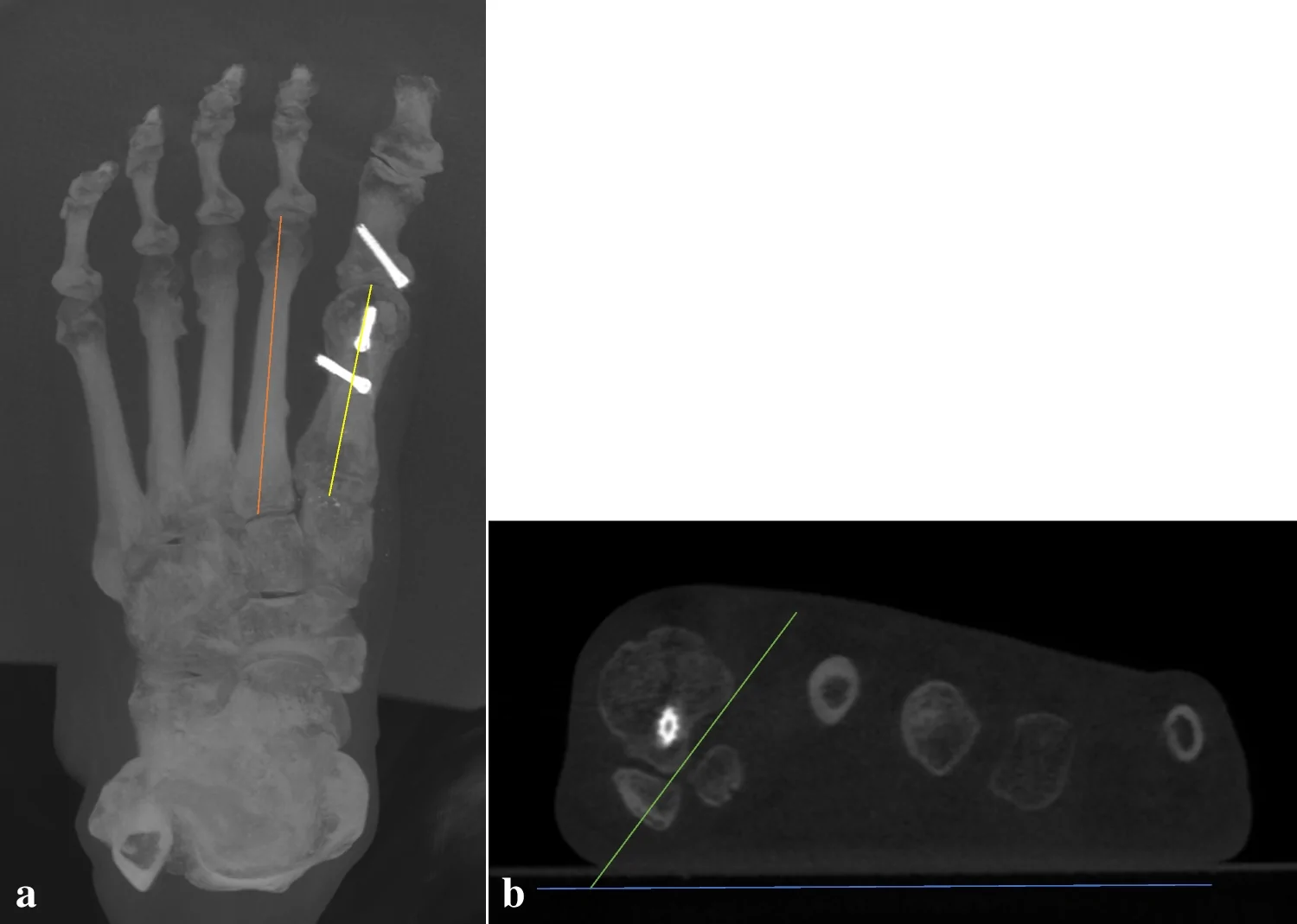
Rotational malunion following Scarf osteotomy. **a** Three-dimensional volume-rendered WBCT image and **b** coronal WBCT reconstruction of the left foot in a 49-year-old woman demonstrating rotational malunion of the first metatarsal following Scarf osteotomy. Although the intermetatarsal angle appears satisfactorily corrected on **a **the axial plane, **b **coronal analysis demonstrates marked residual pronation of the first metatarsal, with a pronation angle of 57°, far exceeding both physiological values (approximately 2°) and those typically reported in hallux valgus (approximately 6°). The rotational deformity is associated with metatarsosesamoid incongruity and lateral displacement of the sesamoid complex, consistent with persistent sesamoid subluxation. This case illustrates the added value of WBCT for detecting postoperative rotational malalignment following hallux valgus surgery. Compared with conventional radiography and non-weight-bearing CT, WBCT enables three-dimensional assessment of first-ray metatarsal rotation and metatarsosesamoid alignment under physiological loading conditions. Rotational deformities may be underestimated on conventional imaging, particularly when satisfactory correction of the intermetatarsal angle masks persistent coronal malrotation. Failure to assess the coronal rotational component may therefore lead to a falsely reassuring interpretation of surgical correction (false-negative assessment). Conversely, suspected rotational abnormalities should be confirmed by multiplanar WBCT analysis to avoid overestimation related to projectional effects or patient positioning (false-positive assessment)

Troughing, a characteristic complication of the Scarf osteotomy, refers to cortical impaction with loss of height of the distal fragment and has been reported in up to 35% of cases (Fig. 12) [78]. Weight-bearing radiographs provide the initial assessment, whereas WBCT is superior for evaluating first metatarsal shortening, loss of height, rotational malalignment, and sesamoid position, abnormalities that are frequently underestimated on conventional radiographs [79] (Fig. 13). Comparison with previous postoperative examinations is essential for assessing the progression of these deformities. The radiologist should accurately characterize the type of malunion and its biomechanical consequences in order to guide subsequent clinical management.

### Metatarsophalangeal joint osteoarthritis

First MTP joint osteoarthritis is a late complication of HV surgery, resulting from persistent joint incongruity, chronic biomechanical imbalance, or, less commonly, avascular necrosis of the first metatarsal head.

Weight-bearing radiographs are the first-line imaging modality. Typical findings include joint space narrowing, dorsal osteophyte formation, subchondral sclerosis, and, in advanced stages, loss of joint congruity consistent with postoperative hallux rigidus.

WBCT provides a more detailed assessment of multiplanar alignment, residual joint incongruity, and areas of abnormal load distribution, particularly in the presence of dorsiflexion malunion or persistent first-ray imbalance. MRI is indicated when radiographic findings are inconclusive, allowing early detection of subchondral bone marrow edema, synovitis, and focal chondral defects.

Residual first-ray deformities, particularly dorsiflexion malunion and first metatarsal shortening, promote the development of degenerative joint changes, whereas preservation of the intraoperative vascular supply to the first metatarsal head reduces this risk [80, 81].

The radiologist should accurately determine the severity of osteoarthritis, identify its underlying mechanism—whether related to persistent joint incongruity, abnormal mechanical loading, malunion, or sequelae of avascular necrosis—and assess its functional implications to guide appropriate clinical management.

### Surgical site infections

Postoperative infections following HV surgery may involve the superficial soft tissues, tendon sheaths, the first MTP joint, or, less commonly, the osteotomy site and fixation hardware. Early diagnosis remains challenging because clinical manifestations are often nonspecific and should always be interpreted in conjunction with laboratory findings [82].

**Fig. 14:**
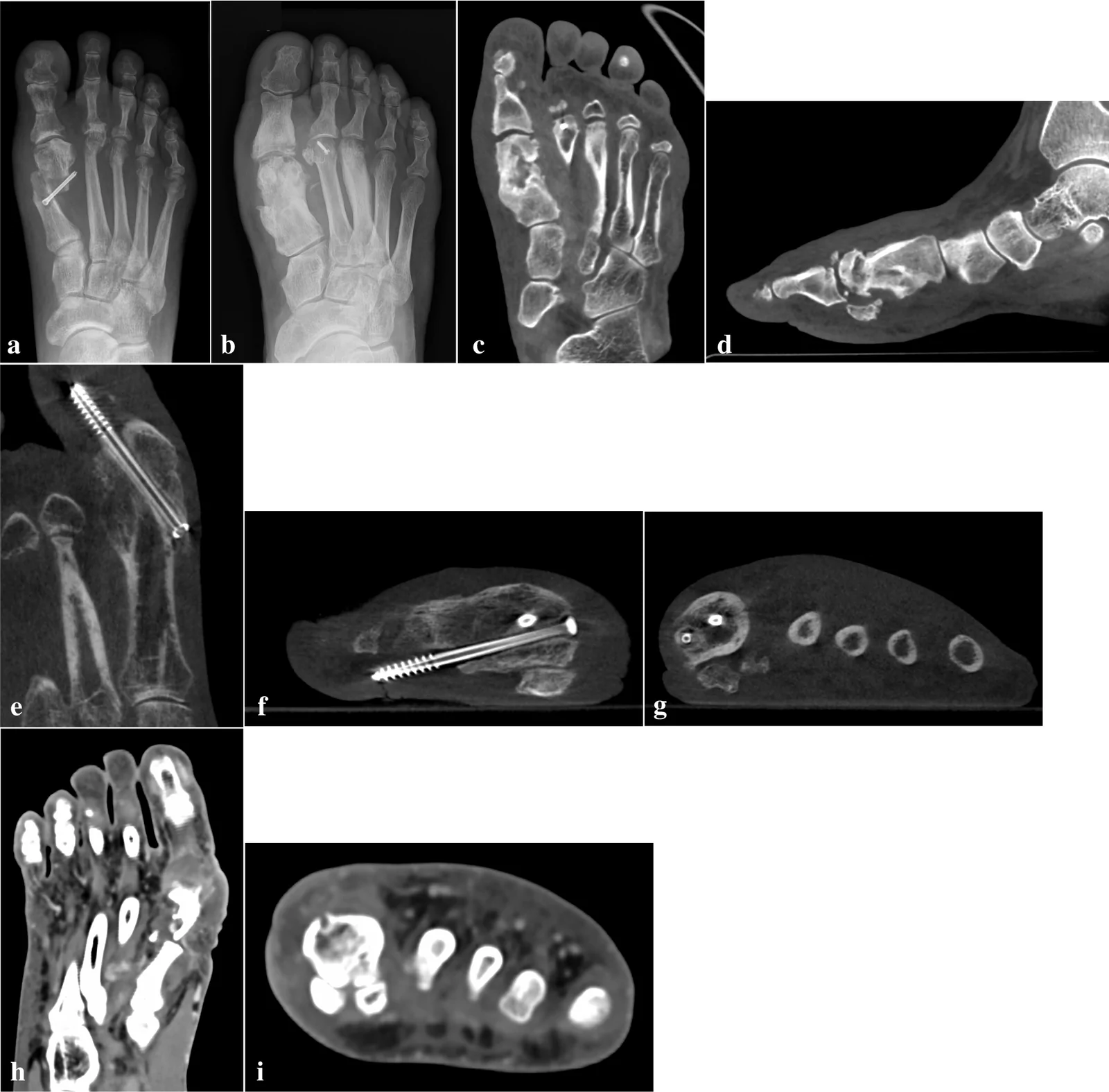
**a** A 59-year-old woman, right foot. Scarf and Akin osteotomies. Weight-bearing anteroposterior (AP) radiograph showing peri-screw lucency at the first MTP joint, suggestive of hardware loosening or migration; in the clinical context, these findings were associated with infection. **b–d** A 51-year-old man, right foot. **b **Weight-bearing AP radiograph, **c **WBCT axial, and **d **sagittal reconstructions. Status post Scarf and Akin osteotomies. Post-correction metatarsalgia with surgical site infection. **e–g** 79-year-old man, left foot. **e **WBCT axial, **f **sagittal, and **g **coronal reconstructions. First MTP arthrodesis using two screws for hallux valgus correction complicated by rotational malposition and postoperative infection. Plantar purulent discharge beneath the hallux with plantar sinus tract adjacent to the medial screw, which protrudes into the soft tissues. Circumferential lucency is present around the screw at the bone–screw interface. **h, i** Left foot. Contrast-enhanced CT with **h** axial and **i** coronal reconstructions in a 52-year-old woman status post Scarf osteotomy, demonstrating dorsomedial collections associated with osseous erosions, consistent with infection

Weight-bearing radiographs are the first-line imaging modality, although their sensitivity is limited during the early stages of infection. Their primary role is to assess first-ray alignment and fixation hardware while identifying late findings such as osteolysis, cortical erosions, or hardware loosening (Fig. 14). Computed tomography provides superior characterization of cortical destruction, osseous sequestra, and deep fluid collections.

Ultrasound is useful for detecting superficial fluid collections and for guiding aspiration or drainage procedures. It also facilitates differentiation between a well-defined abscess and diffuse inflammatory soft-tissue edema.

MRI is the most sensitive modality for evaluating deep postoperative infections, including osteomyelitis, septic arthritis, infectious tenosynovitis, and extension into the surrounding soft tissues. Imaging features most suggestive of infection include replacement of the normal fatty bone marrow signal on T1-weighted images associated with fluid collections, a sinus tract, or extensive soft-tissue involvement, whereas isolated bone marrow edema should be interpreted with caution during the early postoperative period [82–85].

### Nerve injuries and first intermetatarsal space neuralgia after hallux valgus surgery

First intermetatarsal space neuralgia is a postoperative neurological complication of HV surgery, most commonly resulting from intraoperative traction injury or postoperative scar-related irritation of the digital sensory nerve branches. Its reported incidence ranges from 0.4% to 3% across published series [15, 86, 87]. Patients typically present with neuropathic pain, dysesthesia, or allodynia localized to the surgical scar or the first intermetatarsal space.

**Fig. 15:**
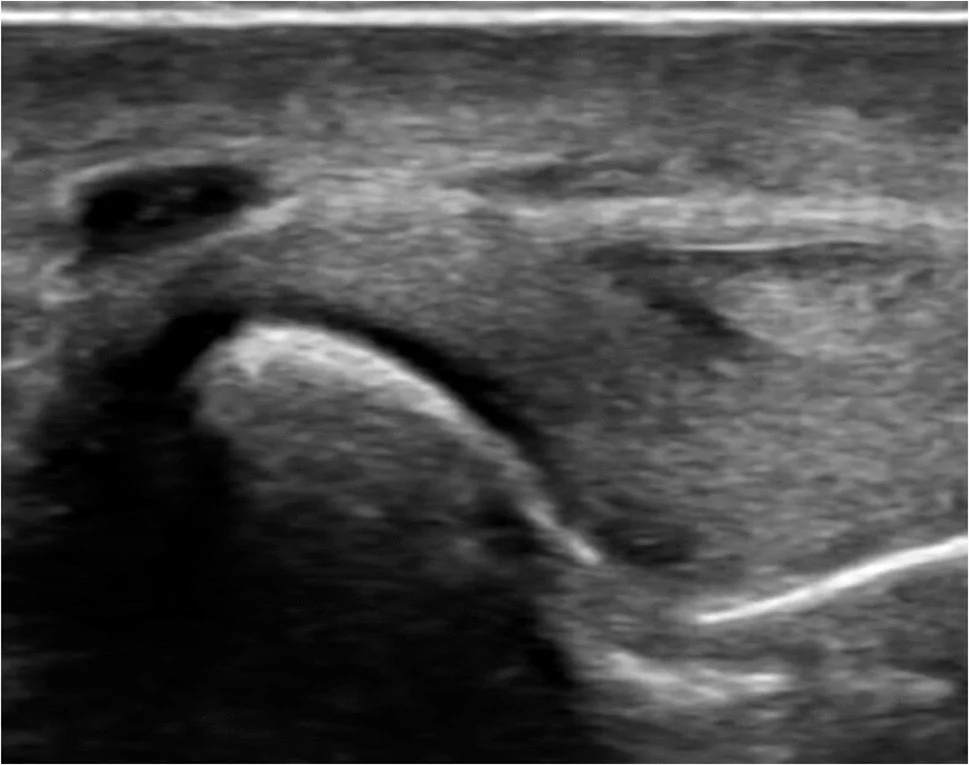
Ultrasound evaluation of a postoperative neuroma after hallux valgus surgery. A 52-year-old woman presented with medial and plantar pain along the first ray following left hallux valgus correction with a Scarf osteotomy. Ultrasound demonstrates a 2 × 3 mm hypoechoic subcutaneous nodule adjacent to the medial aspect of the first metatarsal head, corresponding to the site of maximal tenderness and reproducing the patient’s symptoms during probe palpation. The findings are consistent with a postoperative neuroma involving a digital sensory nerve branch. Targeted ultrasound-guided perineural injection adjacent to the lesion resulted in symptomatic improvement. This case illustrates the value of ultrasound in the evaluation of postoperative neuropathic pain following hallux valgus surgery. Ultrasound enables high-resolution assessment of the superficial digital sensory nerve branches and dynamic correlation with the patient's symptoms, thereby reducing the risk of false-negative diagnoses when neuropathic pain is mistaken for a mechanical complication or postoperative scar-related changes. Conversely, small postoperative hypoechoic scar-related nodules should be interpreted cautiously and correlated with the clinical findings to avoid a false-positive diagnosis of a neuroma

Ultrasound is the first-line imaging modality, allowing visualization of nerve thickening, fascicular disorganization, or an amputation neuroma, which typically appears as a hypoechoic lesion and may demonstrate increased vascularity on Doppler imaging. Ultrasound also enables detailed evaluation of the digital sensory nerve branches and provides real-time guidance for diagnostic or therapeutic interventional procedures (Fig. 15).

MRI is indicated when the diagnosis remains uncertain or in more complex cases. MRI typically demonstrates increased T2 signal intensity within the affected nerve together with adjacent postoperative scar tissue and is also valuable for differentiating neuropathic complications from infection or mechanical postoperative abnormalities.

### Postoperative complications in hallux valgus with metatarsus adductus and juvenile hallux valgus

#### Hallux valgus associated with metatarsus adductus

In HV associated with metatarsus adductus, postoperative complications most often reflect inadequate correction of the overall forefoot alignment rather than an isolated abnormality of the first ray. Weight-bearing radiographs allow identification of incomplete correction or recurrent deformity but may underestimate rotational and multiplanar malalignment. WBCT provides comprehensive three-dimensional assessment of the metatarsals and first ray, making it particularly valuable for planning revision surgery [26]. A common interpretative pitfall is to consider correction of the first-ray satisfactory despite persistent global forefoot malalignment.

#### Juvenile hallux valgus

In juvenile HV, postoperative imaging interpretation must account for the patient’s residual skeletal growth. Recurrent deformity remains the most common postoperative complication and is primarily assessed through serial weight-bearing radiographs demonstrating a progressive increase in the HVA and IMA, together with secondary loss of correction [29]. Other postoperative complications are similar to those observed in adults but should be interpreted in the context of ongoing skeletal growth and physiological osseous remodeling [88–90]. Longitudinal imaging follow-up is essential to distinguish progressive recurrent deformity from growth-related changes and to identify early biomechanical imbalance.

## Weight-bearing computed tomography after hallux valgus surgery: toward a three-dimensional assessment of surgical correction

HV is a three-dimensional deformity of the first ray characterized by lateral deviation of the hallux valgus, medial deviation of the first metatarsal, and pronation of the first ray, with progressive disruption of the metatarsosesamoid complex and medial column stability. Although weight-bearing radiographs remain the reference standard for assessing the HVA, IMA, DMAA, and sesamoid position, their two-dimensional nature limits evaluation of rotational deformities and underestimates the true three-dimensional complexity of the deformity [12, 91–94]. WBCT enables three-dimensional assessment under physiological loading conditions and has substantially improved the understanding of HV by incorporating first metatarsal pronation, abnormalities of the sesamoid complex, and first TMT joint instability into deformity analysis [92, 95, 96].

WBCT is based on volumetric acquisition of the foot in the standing position using either cone-beam CT or dedicated extremity CT systems, with multiplanar and three-dimensional reconstructions [15, 95, 96]. Unlike conventional CT performed in the supine position, WBCT reproduces physiological loading conditions and provides a more accurate evaluation of osseous alignment, joint relationships, and first-ray biomechanics [15, 97–99]. In the postoperative setting, indications include clinicoradiographic discordance, suspected recurrent deformity or incomplete correction, assessment of residual first metatarsal pronation, evaluation of the sesamoid complex, and investigation of persistent pain before revision surgery. The main clinical applications, added value, and limitations of WBCT after HV surgery are summarized in Table 4.

**Table 4:** Clinical applications, strengths and limitations of WBCT after hallux valgus surgery

Clinical situation	WBCT contribution	Added value over weight-bearing radiographs	Main limitations	Impact on patient management
Routine postoperative assessment	Three-dimensional evaluation of first-ray alignment, osteotomy position and hardware	Detects rotational abnormalities not visible on radiographs	Not recommended routinely after uncomplicated surgery	Complements radiographs in selected patients
Suspected recurrent hallux valgus	Assessment of residual metatarsal pronation, sesamoid malalignment and multiplanar recurrence	Identifies persistent rotational deformity despite acceptable HVA and IMA	Limited availability	Guides revision surgical planning
Persistent postoperative pain	Evaluation of osteotomy healing, hardware position, malunion and occult osseous abnormalities	Better assessment of complex osseous causes of pain	Less informative for isolated soft-tissue abnormalities	Helps identify mechanical causes requiring treatment
Assessment after Lapidus arthrodesis	Evaluation of TMT fusion, first-ray alignment, medial column stability and rotational correction	Simultaneous assessment of fusion and rotational correction under physiological loading	Interpretation requires dedicated expertise	Confirms successful correction or identifies residual deformity
Sesamoid complex evaluation	Quantification of sesamoid reduction and rotation	More accurate assessment of sesamoid alignment than radiographs	No universally accepted measurement method	Helps explain persistent symptoms or recurrence
Rotational deformity analysis	Measurement of metatarsal pronation (MPA, alpha angle) and segmental rotation	True three-dimensional evaluation of first-ray rotation	Lack of standardized international measurement protocol	Improves understanding of correction quality
Assessment before revision surgery	Comprehensive evaluation of multiplanar deformity, residual malalignment and bone healing	Provides information unavailable on conventional imaging	Higher cost and limited accessibility	Facilitates surgical decision-making
Radiation considerations	Low-dose cone-beam acquisition under weight-bearing conditions	Lower radiation than conventional CT and may replace multiple imaging examinations	Dose depends on acquisition protocol	Supports ALARA-compliant imaging strategy

WBCT should be considered a complementary imaging modality to weight-bearing radiographs, particularly when rotational deformity, persistent symptoms, recurrence, or revision surgery is suspected

*WBCT* weight-bearing computed tomography, *HVA* hallux valgus angle, *IMA* intermetatarsal angle, *MPA* metatarsal pronation angle, *TMT* tarsometatarsal

The principal advantage of WBCT lies in its ability to provide a comprehensive three-dimensional assessment of the first ray under physiological loading, allowing simultaneous evaluation of angular, rotational, and joint stability parameters [13]. Following osteotomy or Lapidus arthrodesis, WBCT enables assessment of correction of first metatarsal pronation, restoration of sesamoid alignment, medial column stability, and surgery-induced rotational changes [100–104]. It may also reveal persistent malalignment that is not apparent on conventional radiographs despite apparently satisfactory correction of standard angular measurements.

Rotational assessment is primarily based on two parameters. The alpha angle is measured on coronal reconstructions at the level of the first metatarsal head and is defined as the angle between the distal first metatarsal axis and a vertical reference line, primarily reflecting distal metatarsal torsion. The metatarsal pronation angle (MPA), calculated from the orientation of the sesamoid facets relative to the weight-bearing plane, reflects global first-ray pronation under physiological loading. The MPA has demonstrated greater robustness and reproducibility than the alpha angle [91, 92, 105]. As a practical reference, an alpha angle ≥ 15.8° suggests pathological pronation, whereas the MPA ranges from 0 to 2° in asymptomatic individuals and exceeds 10° in cases of significant first-ray pronation [91, 92, 105, 106]. WBCT also enables reproducible assessment of sesamoid rotation, defined by the orientation of the sesamoids’ long axis in the coronal plane, a parameter that correlates strongly with the severity of HV deformity [107].

**Fig. 16:**
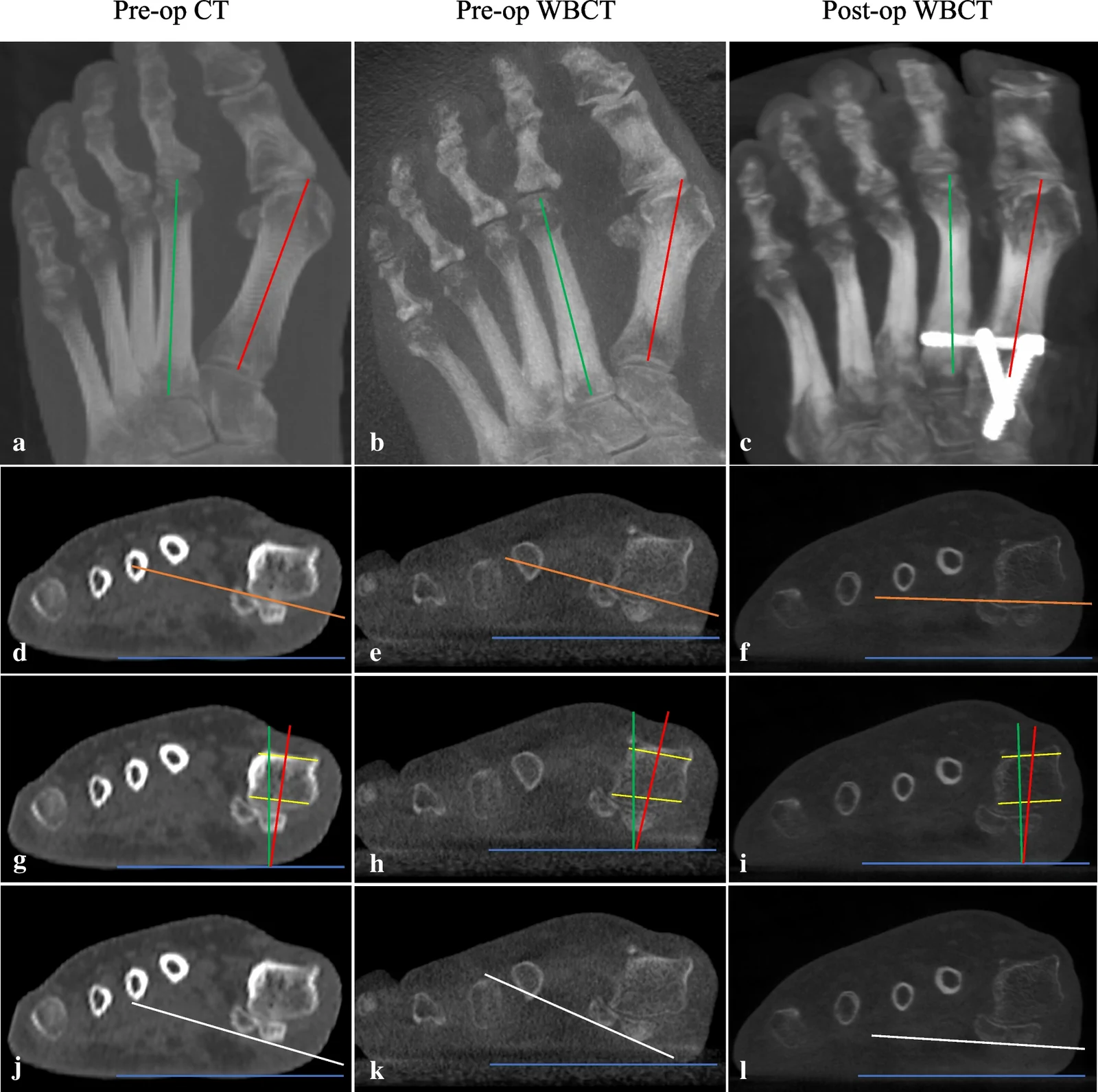
Comparison of conventional non-weight-bearing CT, preoperative WBCT, and postoperative WBCT in the same patient, illustrating the added value of WBCT for assessing the three-dimensional hallux valgus deformity and its surgical correction. The intermetatarsal angle (IMA) measures 19° on the preoperative non-weight-bearing CT (**a**), 21° on the preoperative WBCT (**b**), and 11° on the postoperative WBCT (**c**). The metatarsal pronation angle (MPA) measures 10° on the preoperative non-weight-bearing CT (**d**), 15° on the preoperative WBCT (**e**), and 2° on the postoperative WBCT (**f**). The alpha angle measures 9° on the preoperative non-weight-bearing CT (**g**), 16° on the preoperative WBCT (**h**), and 8° on the postoperative WBCT (**i**). The sesamoid rotation angle measures 17° on the preoperative non-weight-bearing CT (**j**), 25° on the preoperative WBCT (**k**), and 6° on the postoperative WBCT (**l**). Compared with conventional non-weight-bearing CT performed in the same patient, WBCT better demonstrates the three-dimensional severity of the deformity, particularly its rotational component, and provides a more comprehensive assessment of postoperative correction. This comparison highlights the potential for false-negative interpretation when conventional CT is used alone, as the absence of physiological loading may underestimate first metatarsal pronation and sesamoid rotation. Conversely, differences between the two imaging modalities should be interpreted cautiously because variations in patient positioning and the current lack of complete standardization of rotational measurements may result in overestimation of some rotational abnormalities (false-positive interpretation). Overall, WBCT provides a comprehensive three-dimensional evaluation of first-ray alignment under physiological loading, yielding complementary information that is essential for characterizing the initial deformity and assessing the quality of surgical correction

Figure 16 illustrates the added value of WBCT compared with conventional non-weight-bearing CT in the same patient. Whereas conventional CT frequently underestimates the rotational component of the deformity, WBCT clearly demonstrates first metatarsal pronation and sesamoid rotation, thereby providing a more accurate three-dimensional evaluation of HV deformity and the adequacy of surgical correction.

Beyond global assessment of the first ray, WBCT also enables segmental evaluation of rotational alignment throughout the medial column.

**Fig. 17:**
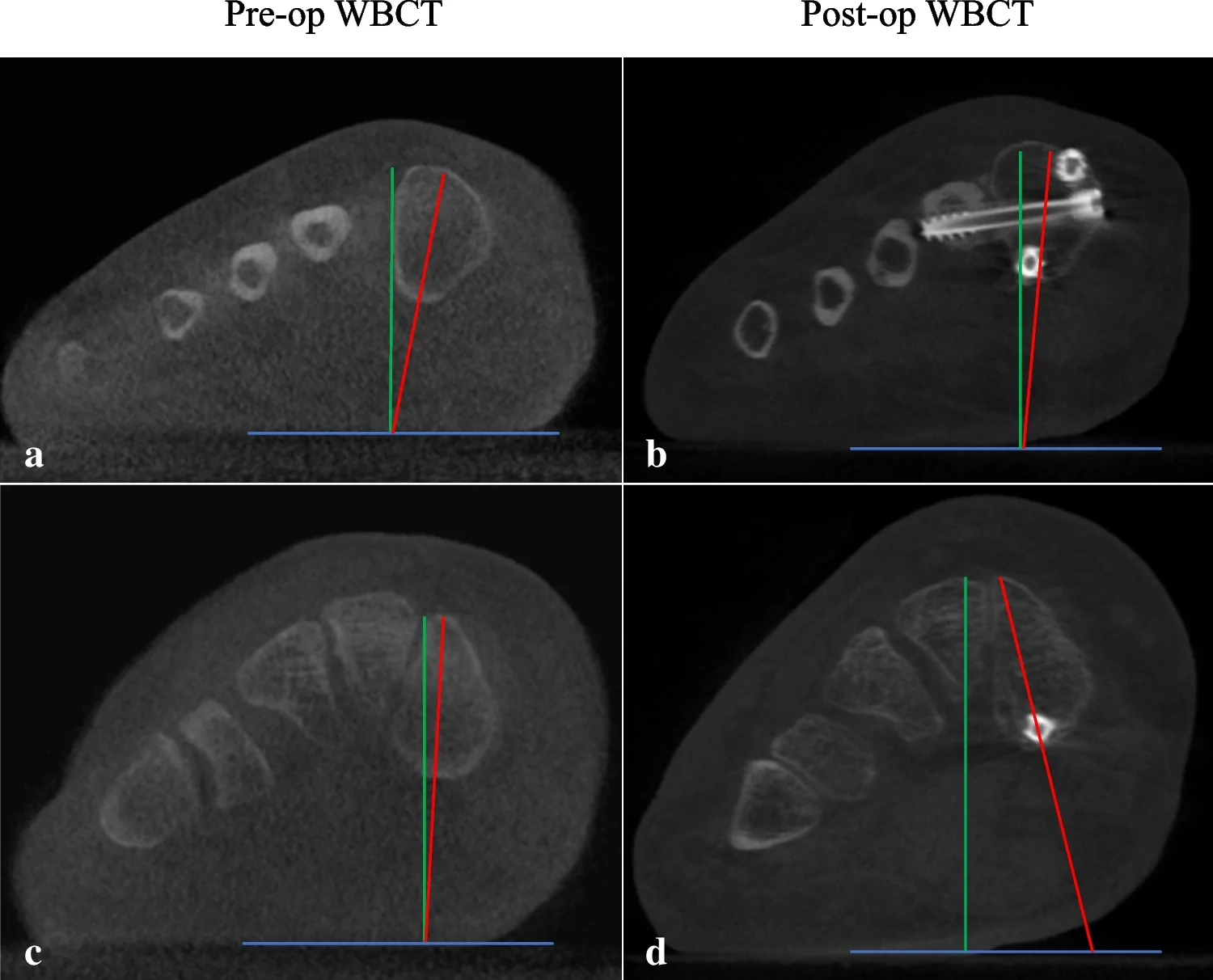
Three-dimensional assessment of first-ray rotation using WBCT before and after surgical correction of hallux valgus with Lapidus arthrodesis. Segmental rotational measurements of the medial column obtained from weight-bearing WBCT acquisitions. **a** Preoperative rotation of the base of the first metatarsal (M1), measuring 13°, consistent with first-ray pronation. **b** Postoperative rotation of the base of the M1, measuring 5°, demonstrating correction of first-ray pronation. **c** Preoperative rotation of the medial cuneiform (C1), showing 3° of pronation. **d** Postoperative rotation of the C1, showing 10° of supination, illustrating rotational realignment of the medial column. This case demonstrates the three-dimensional rotational changes of the medial column following surgical correction of hallux valgus, with reduction of first metatarsal pronation and segmental rotational realignment under physiological loading. It highlights the added value of WBCT over conventional non-weight-bearing CT by enabling assessment of segmental rotational changes within the medial column under physiological loading conditions, which cannot be reliably evaluated on conventional CT performed in the supine position. Segmental rotational analysis is particularly valuable following Lapidus arthrodesis, where successful correction depends not only on reduction of the intermetatarsal angle but also on restoration of the rotational alignment of the first ray. Failure to evaluate this three-dimensional component may lead to a falsely reassuring interpretation of satisfactory correction despite persistent residual pronation (false-negative assessment). However, rotational measurements should be interpreted with caution because of the current limitations of WBCT, including the lack of universal consensus regarding anatomical landmarks and measurement techniques, as well as reported interobserver variability related to reconstruction methods. Multiplanar assessment and correlation with the clinical findings remain essential to avoid overestimation of rotational abnormalities (false-positive assessment)

In the case illustrated in Fig. 17, reduction of rotational deformity at the base of the first metatarsal, together with rotational reorientation of the medial cuneiform toward supination following Lapidus arthrodesis, demonstrates the ability of WBCT to objectively quantify segmental rotational changes induced by surgery. These three-dimensional findings, which are essential for evaluating the quality of surgical correction, cannot be reliably assessed using conventional non-weight-bearing CT.

WBCT may also improve the efficiency of the diagnostic pathway while reducing overall radiation exposure when it replaces strategies combining repeated radiographs and conventional CT. In a cohort of 11,009 examinations performed in 4987 patients, de Cesar Netto et al. reported a significant 10% reduction in the mean annual effective radiation dose (4.3 µSv vs. 4.8 µSv; *p* = 0.01), attributable to replacement of repeated radiographic series (approximately 1.4 µSv) and reduced use of conventional CT (approximately 25 µSv), with bilateral WBCT delivering an effective dose of approximately 4.2 µSv [99]. Phantom studies have reported effective doses ranging from 1.9 to 14.3 µSv depending on acquisition protocols, values that are consistently lower than multidetector CT and comparable to, or lower than, a complete conventional radiographic series when WBCT is used as a single replacement modality, in accordance with the ALARA principle [107].

Current limitations of WBCT include its limited availability, higher cost, and the need for dedicated expertise. Interpretation is further constrained by the lack of standardized methods for rotational and segmental measurements, as no international consensus has yet established a uniform measurement protocol. Although overall reproducibility is good, interobserver variability persists depending on the anatomical landmarks selected and the reconstruction techniques employed [13, 91, 92, 105, 106, 108]. Moreover, three-dimensional analysis remains more time-consuming than conventional imaging and requires a specific learning curve for accurate interpretation.

## Conclusion

HV is a complex three-dimensional deformity requiring individualized surgical management based on comprehensive weight-bearing radioclinical assessment. Postoperative imaging is essential for evaluating correction, osseous healing, and complications. WBCT represents a major advancement in postoperative assessment by enabling three-dimensional analysis of surgical correction under weight-bearing conditions, providing a more accurate evaluation of first-ray alignment and its rotational components. Its integration into the imaging strategy enhances the understanding of postoperative anatomical and biomechanical outcomes and contributes to a more comprehensive assessment of the achieved corrections. A thorough knowledge of expected postoperative findings and potential complications remains essential to optimize radiological interpretation and guide patient management.

## Data Availability

Not applicable.

## Abbreviations

DMAA: Distal metatarsal articular angle
HV: Hallux valgus
HVA: Hallux valgus angle
IMA: Intermetatarsal angle
MTP: Metatarsophalangeal
MRI: Magnetic resonance imaging
TMT: Tarsometatarsal
WBCT: Weight-bearing computed tomography
